# MXene: fundamentals to applications in electrochemical energy storage

**DOI:** 10.1186/s11671-023-03786-9

**Published:** 2023-02-03

**Authors:** Daniel Nframah Ampong, Emmanuel Agyekum, Frank Ofori Agyemang, Kwadwo Mensah-Darkwa, Anthony Andrews, Anuj Kumar, Ram K. Gupta

**Affiliations:** 1 Department of Materials Engineering, College of Engineering, Kwame Nkrumah University of Science and Technology, Kumasi, Ghana; 2 Department of Material Science and Engineering, Hohai University, Nanjing, China; 3 Nano-Technology Research Laboratory, Department of Chemistry, GLA University, Mathura, Uttar Pradesh 281406 India; 4 National Institute for Materials Advancement, Pittsburg State University, Pittsburg, KS 66762 USA; 5 Department of Chemistry, Pittsburg State University, Pittsburg, KS 66762 USA

**Keywords:** MXene, MAX phases, Intercalation, Surface terminations, Electrochemical energy storage

## Abstract

A new, sizable family of 2D transition metal carbonitrides, carbides, and nitrides known as MXenes has attracted a lot of attention in recent years. This is because MXenes exhibit a variety of intriguing physical, chemical, mechanical, and electrochemical characteristics that are closely linked to the wide variety of their surface terminations and elemental compositions. Particularly, MXenes are readily converted into composites with materials including oxides, polymers, and CNTs, which makes it possible to modify their characteristics for a variety of uses. MXenes and MXene-based composites have demonstrated tremendous promise in environmental applications due to their excellent reducibility, conductivity, and biocompatibility, in addition to their well-known rise to prominence as electrode materials in the energy storage sector. The remarkable characteristics of 2D MXene, including high conductivity, high specific surface area, and enhanced hydrophilicity, account for the increasing prominence of its use in storage devices. In this review, we highlight the most recent developments in the use of MXenes and MXene-based composites for electrochemical energy storage while summarizing their synthesis and characteristics. Key attention is paid to applications in supercapacitors, batteries, and their flexible components. Future research challenges and perspectives are also described.

## Introduction

**Fig. 1:**
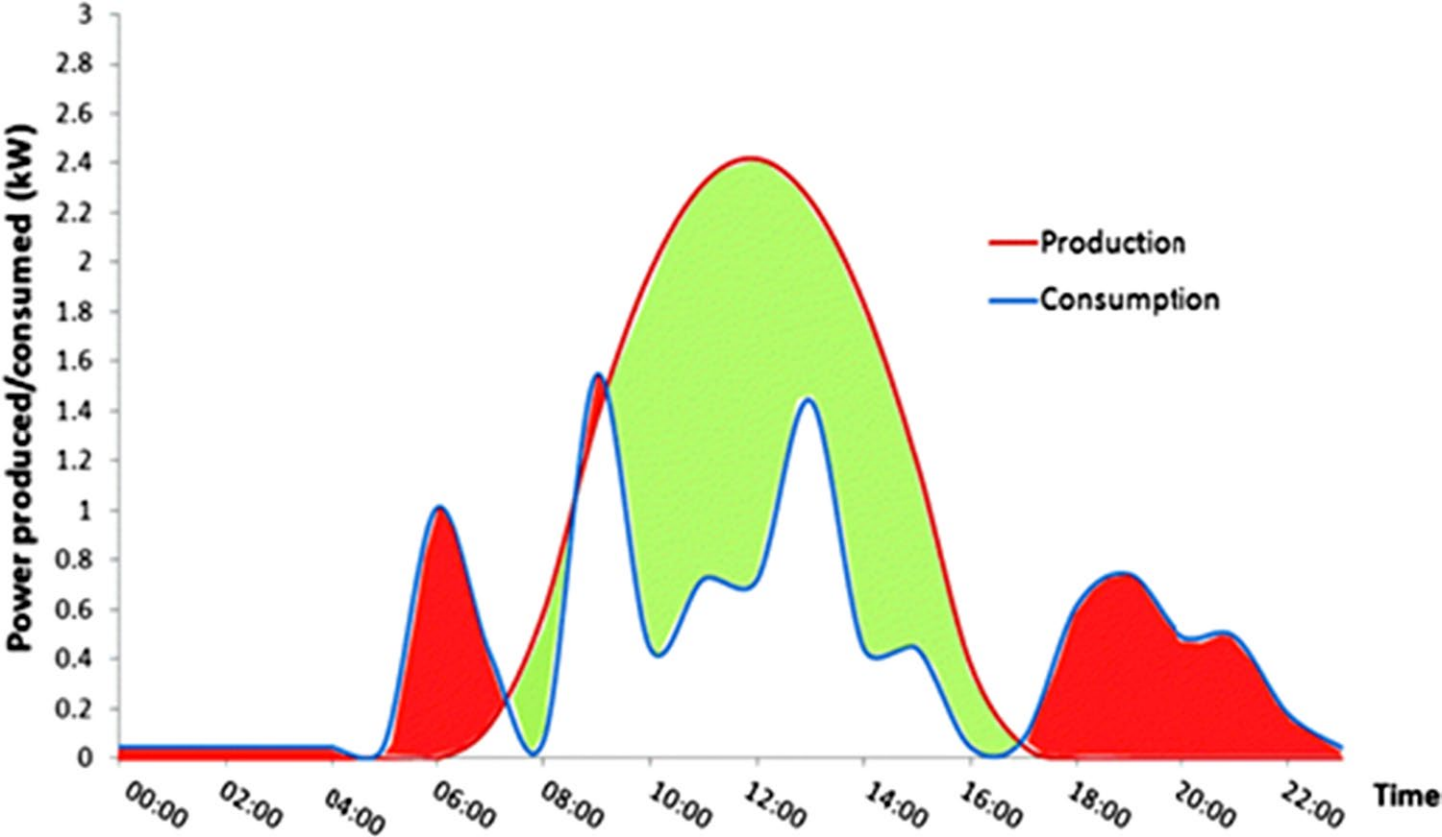
Average electricity generation and consumption from renewable energy. Adapted from reference [6], Copyright (2017) by the authors. Licensee MDPI, Basel, Switzerland. This article is an open-access article distributed under the terms and conditions of the Creative Commons Attribution (CC BY) license

The two most important issues to be dealt with as quickly as possible to stop global climate change are energy and the environment. It should be accorded the highest priority because these are interconnected. Since fossil fuel burning accounts for the greater portion of air pollution and subsequent global warming in today's globe, clean energy sources are still being sought after [1–3]. One of the acknowledged causes for the search for clean energy technologies is the current shift in consumer desire for electric automobiles, smart devices, monitors, the Internet of things (IoT), etc. [4, 5]. Some of the clean energy sources are solar, wind, biomass, geothermal, etc. Among them, solar energy has been widely utilized for electricity supply. The output of the energy varies greatly depending on factors like the hour of the day and the season due to the intermittency of renewable energy. To use the energy that is produced, especially during peak hours, it must be stored. A predicted power generation and consumption curve for renewable energy for a particular season is shown in Fig. 1. From the figure, the amount of electricity generated in a given time (green region) is greater than what the load needs. In this case, the excess energy needs to be stored with the intervention of a storage device [6]. Both for mobile and stationary applications, the selection criteria for renewable energy storage options are still up for debate.

**Fig. 2:**
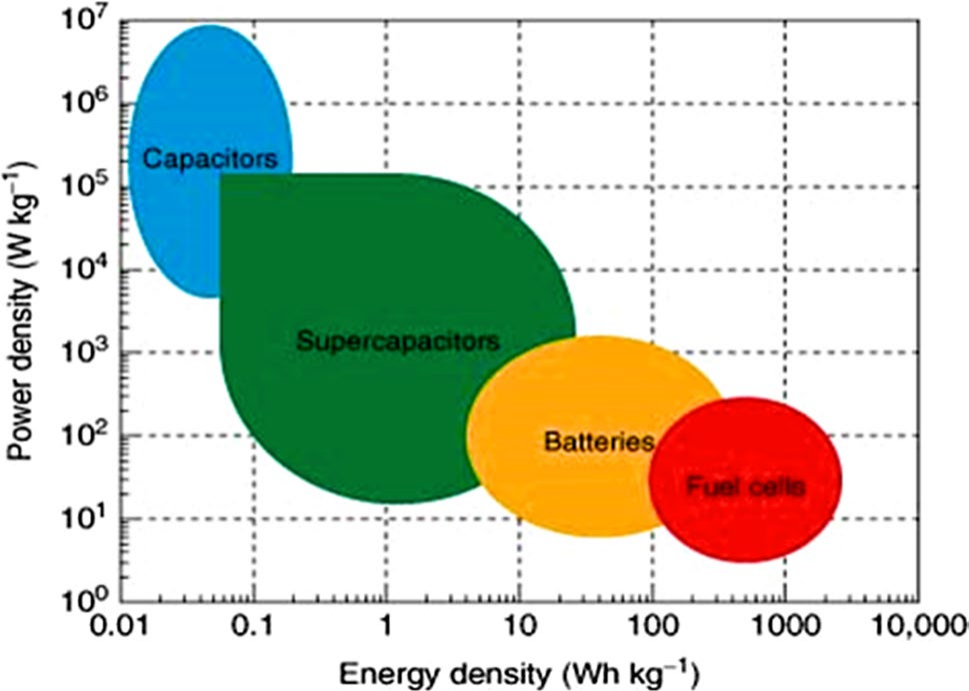
Ragone plot for the various energy storage and conversion devices. Adapted with permission [8], Copyright (2015), John Wiley and Sons

Although conventional capacitors are attractive options for the said applications, there are still concerns about the resources' availability, durability, scalability, and ability to be recycled after decomposition. Some potential electrochemical energy storage (EES) technologies are the supercapacitor (SC) and batteries, which can address or support these problems when used in conjunction with other sustainable energy sources. While SCs can produce high power densities, good cycle rates, low self-discharge, and a wide temperature range, they suffer in the area of low energy density, as shown in Fig. 2. Batteries, on the other hand, are one of the main sources of available electrochemical conversion and storage devices with high energy densities but lacking in terms of self-discharge, charging time, life cycle, temperature tolerance, and various risks in transport applications [7, 8].

One of the most essential components of these EES devices is the electrode. The effectiveness of the electrodes affects the device's performance. Any device's effectiveness in real-time applications depends on the stability and building blocks of its material qualities, which can be customized for each application. It is well established that tailoring nanoscale properties have a much greater impact than tailoring bulk size qualities. Depending on their quantum confinement, nanoscale materials can be categorized as zero-dimension (0D) [9, 10], one-dimension (1D) [11], two-dimension (2D) [12, 13], and three-dimension (3D) [14]. 2D materials have received substantial study due to their intriguing features for a variety of applications. The study of 2D materials is a particularly interesting field in the development of new materials, from graphene and its derivatives to dichalcogenides. 2D materials like graphene have developed their own aesthetic and are advancing toward commercialization. Due to their structural characteristics and other benefits, carbon and its derivatives, including carbon nanofibers, carbon nanotubes, and carbon nanospheres, have been used as EES electrodes.

**Fig. 3:**
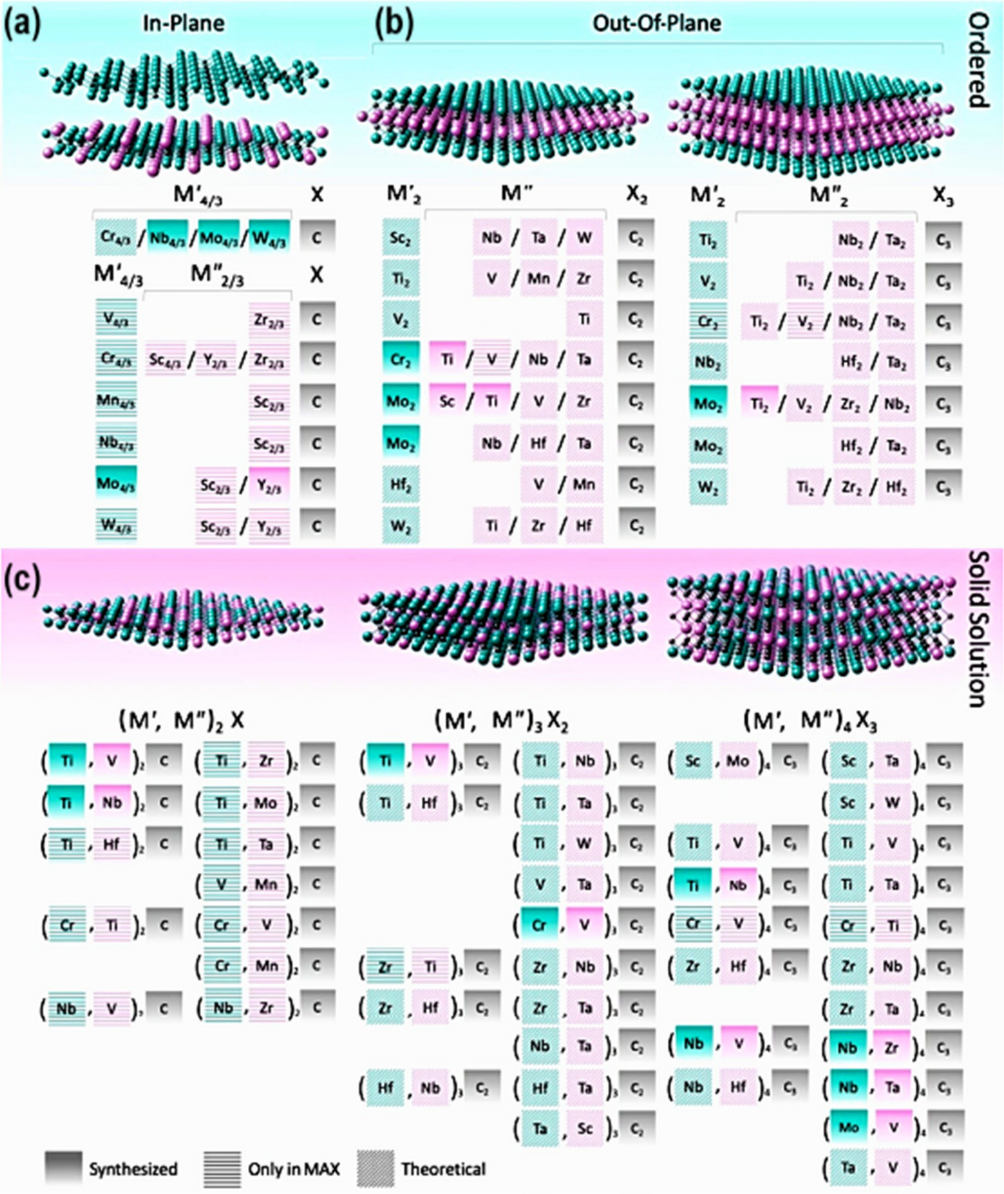
Representation of experimentally as well as theoretically designed DTMs, including **a** in-plane order, and **b** out-of-plane order MXenes. **c** Solid-solution disordered MXenes. Adapted with permission [21], Copyright (2020), Cambridge University Press

MXene has also attracted a lot of research interest as one of the most recent 2D materials. Generally speaking, MXenes were being researched for usage as electrode materials in SCs and lithium-ion batteries (LIBs) because of their remarkable characteristics, including high conductivity, high specific surface area, and enhanced hydrophilicity [15]. The primary method of producing MXenes is by etching the A layers from various MAX phases, comprising ternary nitrides or carbides with the chemical formula M_*n*+1_AX_*n*_ (M denotes an early transition metal, A denotes a group IVA or IIIA element, *X* denotes *C*, and/or N, and *n* = 1, 2, 3, 4). MAX phases have layered hexagonal structures; the M_*n*+1_X_*n*_ units and the A layers are stacked alternately. Since M-X bonds are substantially stronger than M-A bonds, it is possible to selectively chemically etch the A layers without damaging the M-X bonds, leaving behind weakly bonded M_*n*+1_X_*n*_ layers that are easily separable by sonication [16, 17]. There are two types of MXenes: (i) double transition metal (DTM) MXenes, where D stands for two separate transition metals represented by M' and M", and (ii) mono-TM MXenes, where TM stands for only a single type of TM, as in Ti_2_CT_*x*_, V_2_CT_*x*_, Ti_3_C_2_T_*x*_, and Nb_4_C_3_T_*x*_. DTM MXene is identical to mono-TM MXene, except that two TMs of the DTM occupy metal sites instead of just one TM [18]. Based on its structure, DTM MXene may be categorized as (i) ordered (Fig. 3a, b) or (ii) solid solution MXene (Fig. 3c). For the first category, M and M" occupy either in-plane (like Mo_4/3_Y_2/3_CT_*x*_) or out-of-plane (OOP) sites, respectively (like Mo_2_TiC_2_T_*x*_ and Mo_2_Ti_2_C_3_T_*x*_). According to Fig. 3a, in-plane ordered MXene has the formula M_4__/3_M_2/3_XT_*x*_, where M and X are the numbers of M layers, and the TMs are arranged in alternating sites in each M layer. Ordered TM in a different atomic plane and an inner layer of M" metal sandwiched by an exterior layer of M characterize MXenes with the formula M_2_M"X_2_T_*x*_ or M_2_/M"X_3_T_*x*_ [19]. MXene, a solid solution with two TMs, has the formula (M, M)_*n*+1_C_*n*_T_*x*_ (Fig. 3c) [20], similar to (TiV)_2_CT_*x*_, (TiNb)_3_C_2_T_*x*_, and (Nb, Zn)_4_C_3_T_*x*_. All experimentally and theoretically created DTM MXenes up to this point have been carbides. Meanwhile, no reports of DTM MXene (TMCNs or TMCs in particular) have been made. DTM MXene's layered structure is created from its parent MAX phases; therefore, it is fascinating that the composition of DTM MXene can be regulated by the composition of MAX phases. There are more than 20 DTM MXenes made from MAX at the moment (Fig. 3, solid gradient backdrop); however, many of them have not been etched to their matching DTM MXenes (Fig. 3, horizontal lines). Similarly, theoretically predicted DTM MXene (Fig. 3, background diagonal striped line) [21] has not been supported by experimentally produced DTM MXene or their DTM MAX phase precursors. As a new member of the MXene family, M_2_XT_*x*_, the thinnest MXene, has an in-plane-ordered structure where M and M" occupy each M layer (Fig. 3a), where M represents V, Nb, Cr, Mo, W, Mn, and M is Sc, Y, or Zr. Similar to other MXenes, the unique atomic ordering in this form of MXene arises from an in-plane-ordered MAX phase precursor. This is because of four factors: (i) the 2:1 stoichiometric ratio of M and M", (ii) the remarkable difference in atomic radii of M and M", where M" is bigger, (iii) the significant difference in electronegativity of M" and A-layer atoms, and (iv) the selective etching of the Al layer to form an in-plane-ordered DTM MXene. In addition, introducing vacancies causes differences in electrochemical active sites or changes the surface termination quantity and distribution along with M. As an illustration, divacancies cause Mo_4_X_3_CT_*x*_ to have more -F terminations than Mo_2_CT_*x*_. In Mo_4/3_CT_*x*_, the electron shortage for surface termination due to the reduced Mo concentration improves -F termination over = O termination. Electrochemical reactions are set in motion by the more electrically conductive Mo_4/3_CT_*x*_.

Due to the lack of information on the photo/electrocatalytic activity of in-plane-ordered DTM and divacancy MXene, further research into the change-transport kinetics in DTM, as well as the impacts of vacancies and surface termination composition, is warranted. In contrast, MXenes formed using out-of-plane ordered DTM [22] have M metal layers at their core (shown in purple in Fig. 3b), which are sandwiched between layers of M' (shown in green) at their surface. Only Mo_2_ScAlC_2_, Cr_2_VAlC_2_, MoTiAlC_2_, and MoTi_2_AlC_3_ have been produced experimentally as out-of-plane ordered DTM MAX phases. When strictly etched, ordered MAX phases retain the structural ordering that makes them unique, and this is demonstrated in the derivative material MXene. Some out-of-plane ordered DTM MXenes may exhibit semi-conductor or semi-metallic conductivity, while Momo-M, M_3_X_2_T_*x*_, and M_4_X_3_T_*x*_ commonly exhibit polar conductivity (metallic) [22–26].

MXenes have a wide range of interesting electrical, mechanical, magnetic, and electrochemical features thanks to their adaptable chemistries. It is particularly easy for MXenes to build composites with different materials due to their high flexibility, 2D morphologies, and layered architectures. This opens up the possibility of combining the exceptional qualities of several materials in a complementary manner [27]. As a result, not only MXenes but also MXene-based composites have generated a lot of research interest and show tremendous promise for a variety of applications. Due to their good electrochemical activity and high conductivity, MXenes-based materials have been used as high-performance electrode materials for supercapacitors, sodium-ion batteries, and lithium–sulfur batteries [28].

Amazingly, they have recently become even more well known in disciplines relating to the environment. In particular, they have been applied to gas sensors and biosensors that exhibit superb performances [29]. They can remove/reduce contaminants such as organic dyes, heavy metal ions, and eutrophic substances from water [30]. They have also been employed as effective catalysts/co-catalysts for the applications of electro/photocatalytic water splitting and photocatalytic CO_2_ reduction [31]. While the uses of MXenes in environmental applications have been recognized and thoroughly covered in some reviews, the most hopeful developments in MXenes' energy storage applications have not been comprehensively outlined. Although there have been a few reported reviews on MXenes, this work focuses primarily on MXenes and MXene-based composites for electrochemical energy storage applications. In this review, we highlight the most recent developments in the use of MXenes and MXene-based composites for electrochemical energy storage while summarizing their synthesis and characteristics. The most common synthesis techniques, such as the top-down (HF, fluorine-based salt, anhydrous, Lewis etching, etc.) and bottom-up (chemical and physical vapor deposition) approaches, for MXene have been discussed. Also, physical and chemical methods for synthesizing MXene nanocomposite materials have been carefully presented. In addition, the various properties (electronic, mechanical, and electrochemical) and how they can be modified for enhanced storage abilities have been highlighted. Key attention is paid to applications in supercapacitors, batteries, and their flexible components. Future research challenges and perspectives are also described.

## Synthesis of MXenes and their nanocomposites

**Fig. 4:**
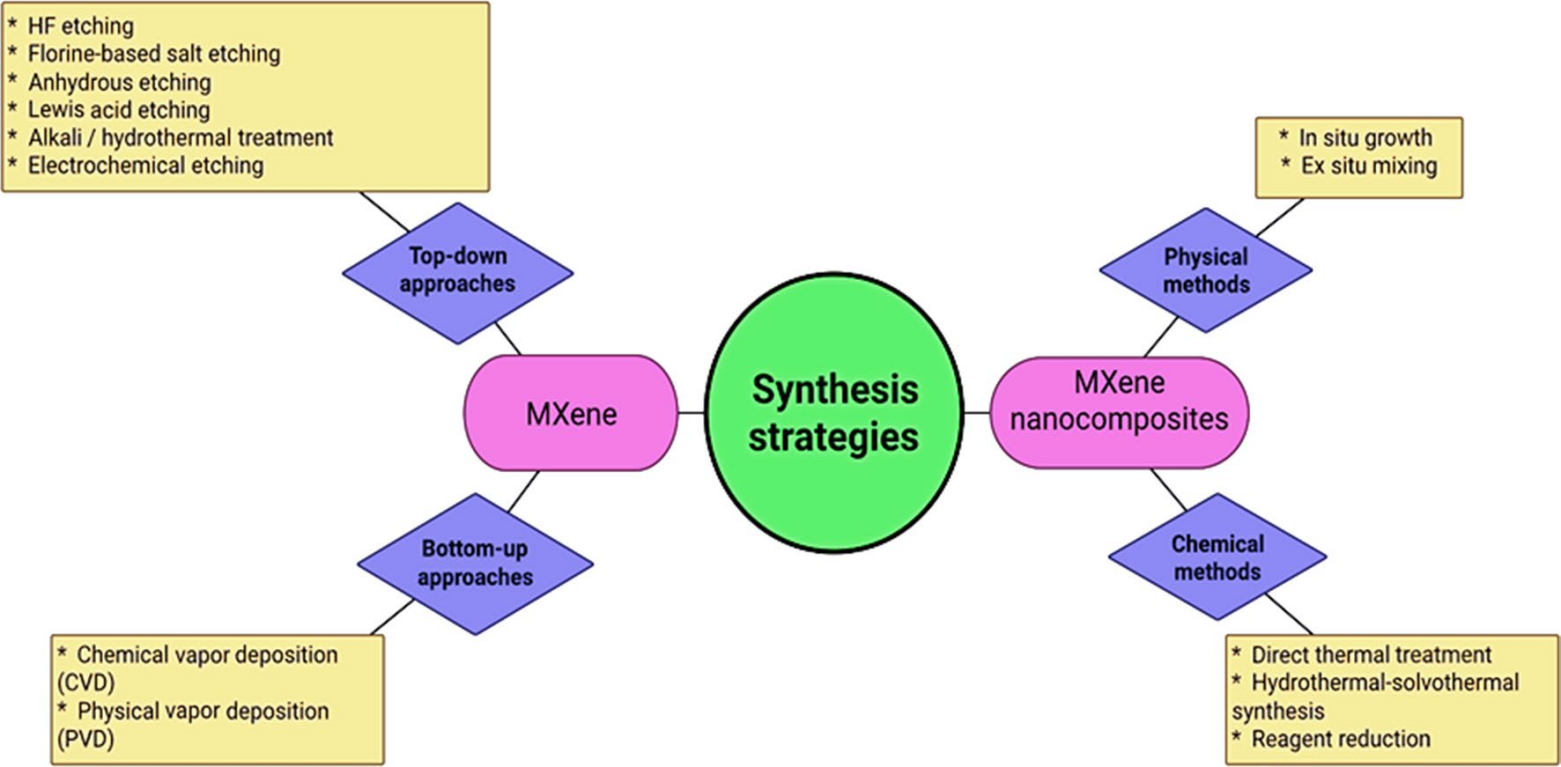
Generalized synthesis strategies for MXenes and their nanocomposites

More than 150 MAX phases, including ordered double intermediate metal structures and solid solutions, have been described in the literature; more than 30 MXenes out of that total have been produced from these phases [32, 33]. Top-down and bottom-up methods are the two primary strategies used for the synthesis of 2D MXenes. In contrast to the bottom-up strategy, which focuses on the development of MXenes from atoms and molecules, the top-down method corresponds to the exfoliation of huge crystal amounts into single-layered MXene sheets [27, 34]. MXene-based nanocomposites have been developed in recent times to overcome the limitations of pure MXene materials. Synthesis strategies for MXenes and their nanocomposites can be categorized as shown in Fig. 4.

### Top-down approach

Selective etching of the A layer in the MAX phases is the standard top-down method for producing MXenes from MAX phases. Before etching, the MAX phase has a three-dimensional (3D) structure, and the etching process can change the MAX phase into MXene, which has a two-dimensional (2D) layered structure [35]. The A elements are etched off once the chemical link between the M and A elements in the MAX phase is broken during the etching process. While the MA bond is metallic in origin, the strong MX bond is a combination of covalent, metal, and ionic bonding [36]. The MX and MA bonds can both be broken at high temperatures, creating a structure that resembles a rock [37]. Additionally, both M and A elements are removed during etching with extremely corrosive chlorine, which results in the formation of carbide derivatives. Therefore, the two etching techniques should be wisely chosen, and a suitable technique should be used to specifically etch out the A element [38]. HF etching, fluorine-based salt etching, anhydrous etching, Lewis acid etching, alkali etching/hydrothermal treatment, and electrochemical etching are some of the etching procedures and agents used in various studies.

#### HF etching

In the first 2D MXene synthesis described by Naguib et al*.* [39], the Al was dissolved to generate Ti_3_C_2_T_*x*_, and the etching was carried out at room temperature on a Ti_3_AlC_2_ MAX phase. HF is thought to be a very selective etching agent. The etching period, HF concentration, MAX phase morphology, particle size, and interatomic interaction strength are all factors that impact the nature of the MXene [16]. Selective HF etching of MXene surfaces results in a variety of potential terminations (OH, F, O, and H). Consequently, the formula M_*n*+1_X_*n*_T_*x*_ is frequently used for MXenes (where T symbolizes the possible surface terminations, e.g., –OH, –F, –O, –H, etc.). Equation (1) describes the reaction pathway for selectively etching A from M_*n*+1_AX_*n*_ phases while the resulting M_*n*+1_X_*n*_ reacts with H_2_O as shown in Eq. (2) and later with HF as indicated in Eq. (3), resulting in the surface -OH or -F functional groups in the resulting MXenes.

1 $${\text{M}}_{n + 1} {\text{AX}}_{n} + 3{\text{HF}} \to {\text{M}}_{n + 1} {\text{X}}_{n} + {\text{AF}}_{3} + 3/2{\text{H}}_{2}$$

2 $${\text{M}}_{n + 1} {\text{X}}_{n} + 2{\text{H}}_{2} {\text{O}} \to {\text{M}}_{n + 1} {\text{X}}_{n} \left( {{\text{OH}}} \right)_{2} + {\text{H}}_{2}$$

3 $${\text{M}}_{n + 1} {\text{X}}_{n} + 2{\text{HF}} \to {\text{M}}_{n + 1} {\text{X}}_{n} {\text{F}}_{2} + {\text{H}}_{2}$$

By raising the etching temperature, the etching time, which is influenced by the strength of the metallic M-A bonding in the MAX phase, can be shortened. Usually, strong HF or a lengthy etching time are required to synthesize MXenes (M_*n*+1_X_*n*_) with a high *n*. For instance, at similar etching conditions, Mo_2_Ti_2_AlC_3_ [40] has a twofold longer etching duration of about 96 h than Mo_2_TiAlC_2_, which was 48 h [22]. MXenes have been discovered to be accessible in non-MAX phases as well. For instance, Meshkian et al*.* [41] obtained Mo_2_C by selectively etching the gallium (Ga) layer from the Mo_2_-Go_2_C MAX phase. Although Mo_2_Go_2_C has a distinct structure from MAX phases, it does have layers of Ga-atoms sandwiched between layers of Mo_2_C. Furthermore, Zr_3_Al_3_C_5_ in the non-MAX phase was reacted with HF, AlF_3_, CH_4_, and Zr_3_C_2_ to selectively remove the Al_3_C_3_ layers, resulting in Zr_3_C_2_. As it is, Zr_3_C_2_ will react with water and HF to produce various surface terminations (–OH, –F, –O, and –H) [42]. Unlike the MAX phase, which has Al layers separating the metal carbide, nitride, or carbon nitride layers, non-MAX phases frequently feature Al-C intercalating layers or units, which result in either M_2_C or M_3_C_2_ MXenes upon etching by the removal of either Al or Al–C. Therefore, non-MAX phases have enormous potential for the synthesis of novel MXene types [43]. Because it is simple to synthesize, and manage in the laboratory, and is inexpensive to prepare, the selective HF-etching approach is the most preferred among researchers. The use of dangerous chemicals, however, is the main disadvantage of this etching technique, which somewhat diminishes its appeal.

#### Fluorine-based salt etching

##### Mono-fluoride salts

To create MAX phases by etching the A-layer, HF-etching uses a mainly hydrated solution with fluoride ions, such as LiF + HCl or HF mixes, as opposed to using straight HF due to its corrosive nature and environmental considerations. The A-atoms are etched as a result of the HCl and LiF interaction, which creates HF in situ [17]. The etching concept of the fluoride salt and strong acid approach is comparable to that of HF etching; however, in the former, metal cations like Li^+^ and Na^+^ as well as water intercalation are present. Since these cations have positive charges while the surface of MXene has a negative charge, they can be injected into the interlayer of MXene to increase the interlayer distance, weakening the contact between MXene nanosheets and minimizing the self-stacking phenomenon of MXene [38]. Additionally, the MXenes do not require any additional delamination procedures to produce single-layer or single-layer flakes. Ghidiu et al*.* [17] etched Ti_3_AlC_2_ with lithium fluoride (LiF) and HCl to produce single-phase Ti_3_C_2_T_*x*_ in high purity. Wang et al*.* [44], who used an ammonium fluoride solution, later disclosed a simpler and safer hydrothermal technique for selectively etching Ti_3_AlC_2_ (NH_4_F). The two instances are depicted in Eqs. (4, 5):

4 $${\text{LiF}} + {\text{HCl}} \to {\text{ HF}} + {\text{LiCl}}$$

5 $${\text{NH}}_{4} {\text{F}} + {\text{H}}_{2} {\text{O}} \to {\text{NH}}_{3} \cdot {\text{H}}_{2} {\text{O}} + {\text{HF}}$$

**Fig. 5:**
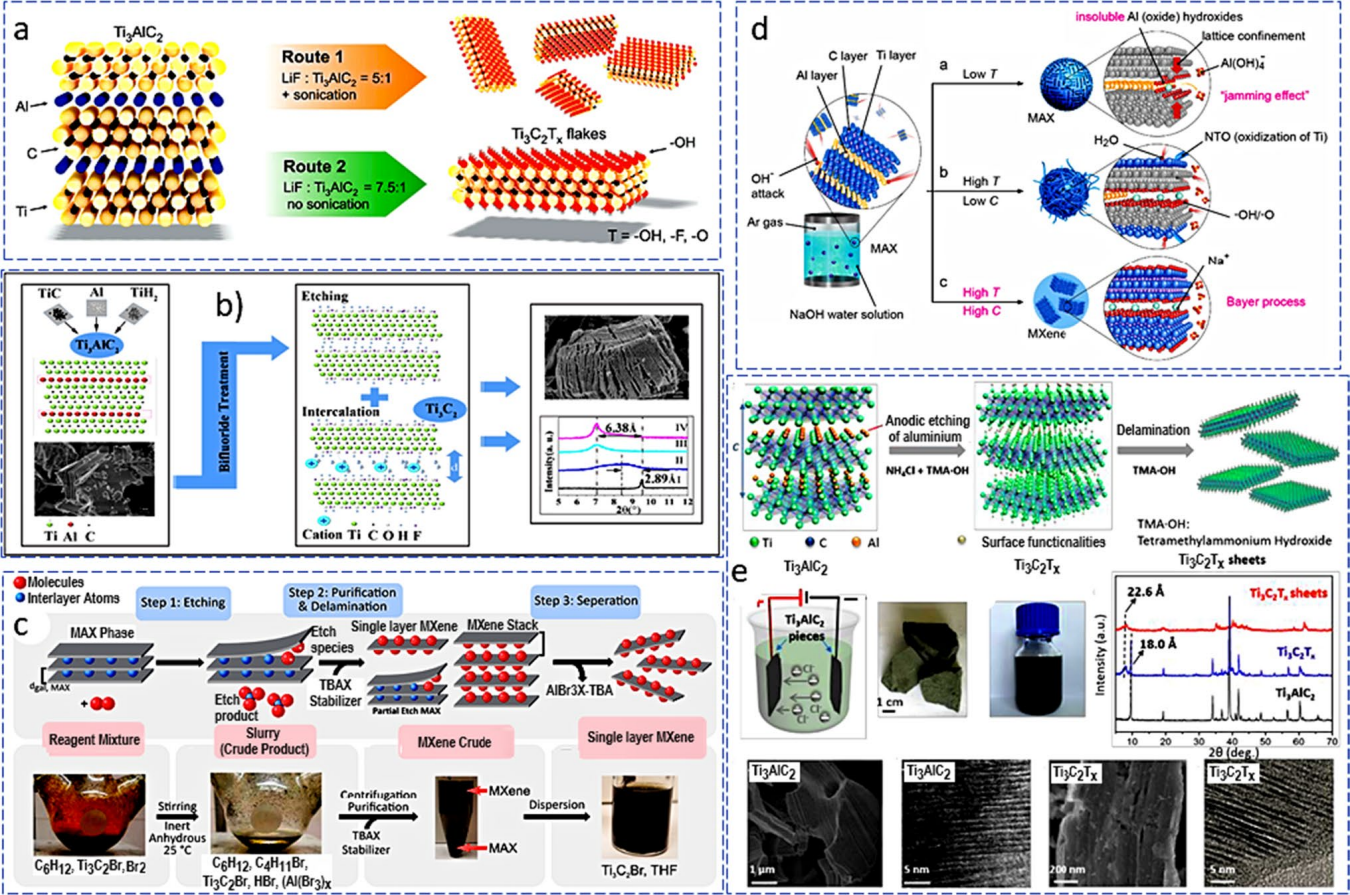
**a** A summary of the schematic preparation of Ti_3_AlC_2_ and Ti_3_C_2_T_*x*_. Adapted with permission [45], Copyright (2016), John Wiley and Sons. **b** A schematic fabrication process and results obtained. Adapted with permission [46], Copyright (2017), Elsevier. **c** Synthesis routes for a deep red solution by the halogen etching of MAX phases. Adapted with permission [50], Copyright (2021), American Chemical Society. **d** A schematic reaction between NaOH water solution and Ti_3_AlC_2_ under various circumstances. Adapted with permission [55], Copyright (2018) John Wiley and Sons. **e** X-ray diffraction patterns, SEM pictures, and cross-sectional HR-TEM images of Ti_3_AlC_2_, Ti_3_C_2_T_*x*_, and Ti_3_C_2_T_*x*_ film from anodic etching of bulk Ti_3_AlC_2_ in a binary aqueous electrolyte. Adapted with permission [56], Copyright (2018), John Wiley and Sons

As opposed to the accordion-like lamellae morphology of MXenes obtained through HF etching, these approaches produce MXene flakes as shown in Fig. 5a. Again, utilizing LiF/Ti_3_AlC_2_ at a concentration ratio of 5:1, followed by sonication, produced small MXene nanosheets, but handshaking for 5 min and increasing the LiF/Ti_3_AlC_2_ concentration ratio to 7.5:1 produced big MXene flakes [45]. Few-layer MXenes or individual-layer MXenes can be produced by adjusting the concentration ratio of LiF/Ti_3_AlC_2_ without the need for additional processing steps like mechanical vibration or ultrasonication [45]. An (NH_4_)_3_AlF_6_ salt was created using NH_4_F and AlF_3_. Single MXene sheets delaminate as a result of the Li^+^ and NH_4_^+^ ions intercalating the layers between the sheets [44]. The use of fluoride-based synthetic processes, however, prevents this promising class of materials from being used in real-world applications.

##### Bi-fluoride salts

To create Ti_3_C_2_ MXenes more safely, the volatile and perilous HF can also be swapped out with the relatively mild bi-fluoride salts: NH_4_HF_2_, NaHF_2_, and KHF_2_ (Fig. 5b) [46]. The overall description of the etching mechanism is shown in Eqs. 6–8:

6 $$2{\text{Ti}}_{3} {\text{AlC}}_{2} + 3{\text{YHF}}_{2} \to {\text{Y}}_{3} {\text{AlF}}_{3} + {\text{AlF}}_{3} + 3/2{\text{H}}_{2} + 2{\text{Ti}}_{3} {\text{C}}_{2}$$

7 $${\text{AlF}}_{3} + x{\text{H}}_{2} {\text{O}} \to {\text{AlF}}_{3} \cdot x{\text{H}}_{2} {\text{O}}$$

8 $${\text{Ti}}_{3} {\text{C}}_{2} + {\text{YHF}}_{2} + {\text{H}}_{2} {\text{O}} \to {\text{Ti}}_{3} {\text{C}}_{2} {\text{F}}_{y} ({\text{OH}})_{z} {\text{Y}}_{m}$$

The process produces Ti_3_C_2_ with many surface termination groups known as Ti_3_C_2_F_*y*_ (OH)_*z*_Y_*m*_. By extending the interlayer gap, the intercalated Y cations (NH_4_^+^, Na^+^, or K^+^) produce MXenes with a 2D flake morphology [47]. Similar to other etching techniques, the resultant MXene product is impacted by variables such as etching time, bi-fluoride concentration, and temperature [48]. However, water is the primary solvent used in the majority of etching techniques, and hydrophilic surface tail groups (T_*x*_) such as -O, -OH, -F, and -Cl are used to functionalize the exposed M-layer faces of etched MAX nanosheets. This restricts the use of MXene in systems with water sensitivity [38].

#### Anhydrous etching

Due to the absence of water, water-free etching has the potential to improve MXenes' electrocatalytic performance. For instance, lithium-ion batteries and sodium-ion batteries with organic electrolytes can be harmed by even small amounts of water [49]. Vaia et al*.* [50] described a productive room-temperature etching technique that produces MXenes from Ti_3_AlC_2_ using halogens (Br_2_, I_2_, ICl, and IBr) in anhydrous conditions (Fig. 5c). The molar ratio of the halogen to the MAX phase, the absolute halogen concentration, the solvent, and the temperature all have a significant role in a radical-mediated reaction. With the help of this etching technique, MXene characteristics can be modified by carefully chosen surface chemistries. Additionally, Natu and co-workers [51] demonstrated that MAX can be etched and delaminated in the absence of water by utilizing organic polar solvents in the presence of ammonium dihydrogen fluoride. They also demonstrated that Ti_3_C_2_T_*z*_ flakes with a lot of fluorine terminations can be made using this etching technique. Further, they showed that Ti_3_C_2_T_*z*_ electrodes etched in propylene carbonate produced Na-ion battery anodes with twice the capacity of electrodes etched in water.

#### Lewis acid etching

Fluorine-based acids and salts can have negative environmental effects when used for etching. Furthermore, extra -F groups on the surface of MXene might negatively impact how well they perform in environmental applications. Recently, Lewis acid etching has been acknowledged as a practical method for creating fluorine-free MXenes. Li et al*.* [52] achieved the creation of Ti_3_C_2_Cl_2_ and Ti_2_CCl_2_ MXene further by reducing Ti_3_ZnC_2_ and Ti_2_ZnC in the molten salt of ZnCl_2_ after successfully preparing a series of MAX phases with Zn by elemental replacement method. Lewis acid, like ZnCl_2_, has a significant acidity when it is molten, and this acidic environment assisted to remove Al from the MAX phase. The main force behind the outward dissemination of Al was the volatility of AlCl_3_. Additionally, diffusion was encouraged by the liquid environment, which helped the replacement reaction to succeed. First, the raw material was prepared using a combination of Ti_3_AlC_2_:ZnCl_2_ = 1:1.5. A simplification of the reaction (Eq. 9) could be used to generalize the development of the Zn-containing MAX phase [52]:

9 $${\text{Ti}}_{3} {\text{AlC}}_{2} + 3/2{\text{ZnCl}}_{2} \to {\text{Ti}}_{3} {\text{ZnC}}_{2} + 1/2{\text{Zn}} + {\text{AlCl}}_{3}$$

An alumina crucible holding the Zn-containing MAX phase was fed into a tube furnace at 550 °C for 5 h while being ventilated with Ar. After the reaction, the product was obtained and dried at 40 °C after being washed with deionized water. This method of etching mechanism was comparable to HF etching, where Zn^2+^ and Cl^−^ in the ZnCl_2_ played roles that were equivalent to H^+^ and F^−^, respectively. Several conventional (aluminum-containing) and unconventional (silicon-, zinc-, and gallium-containing) MAX phases were transformed into comparable MXenes using a new Lewis acid etching technique developed by Li et al*.* [28]. The standard approach used titanium silicon carbide (Ti_3_SiC_2_) as a precursor and copper (II) chloride (CuCl_2_) molten salt as an etchant. The as-prepared Cu particles were later removed from the product by treating it with (NH_4_)_2_S_2_O_8_ (ammonium persulfate) solution. The concluding Ti_3_C_2_T_*x*_ MXene mostly contained O-terminated groups as well as Cl-terminated groups since O-containing groups were introduced during this process. The authors also showed that MXenes produced by this method have the potential to develop into high-rate negative electrode materials [53]

#### Alkali etching or hydrothermal treatment

Although synthesis methods based on HF etching and fluoride salts could successfully remove the "A" layer from the MAX phases, using HF solution (even at low concentrations) is not environmentally friendly. Therefore, the creation of HF-free etching procedures is crucial for both environmental and safety reasons. Recently, high-quality MXene has been produced by investigating alkaline etching of the MAX phases. It is essential to remove dissolved oxygen from DI water by bubbling with Ar gas since MXenes are sensitive to dissolved oxygen. Equations (10) and (11) demonstrate how the MAX phase may be successfully etched by the hydrothermal treatment followed by repeated washing processes while using concentrated NaOH in deaerated water [54]

10 $${\text{Ti}}_{3} {\text{AlC}}_{2} + {\text{OH}}^{ - } + 5{\text{H}}_{2} {\text{O}} \to {\text{Ti}}_{3} {\text{C}}_{2} \left( {{\text{OH}}} \right)_{2} + {\text{Al}}\left( {{\text{OH}}} \right)_{4} + 5/2{\text{H}}_{2}$$

11 $${\text{Ti}}_{3} {\text{AlC}}_{2} + {\text{OH}}^{ - } + 5{\text{H}}_{2} {\text{O}} \to {\text{Ti}}_{3} {\text{C}}_{2} {\text{O}}_{2} + {\text{Al}}\left( {{\text{OH}}} \right)_{4} + 7/2{\text{H}}_{2}$$

As presented in Fig. 5d, Li et al. [55] published a method in 2018 for producing Ti_3_C_2_T_*x*_ MXene using NaOH. Hydroxide anions (OH) target the layers of aluminum in this process, which causes the oxidation of aluminum atoms. Alkali is then added to the produced aluminum hydroxides (Al(OH)_3_), and the exposed Titanium atoms are completed by either OH or O. The process also results in the creation of fresher Al hydroxides, which are prevented from interacting with the OH again because they are contained within the titanium layers' lattice. The problem was resolved by employing a range of hydrothermal temperatures as well as sodium hydroxide water solution concentrations in an Ar atmosphere. The MXenes made using this hydrothermal process have higher OH and O terminations than their HF-etched equivalents, which significantly boosts their supercapacitor efficiency.

**Fig. 6:**
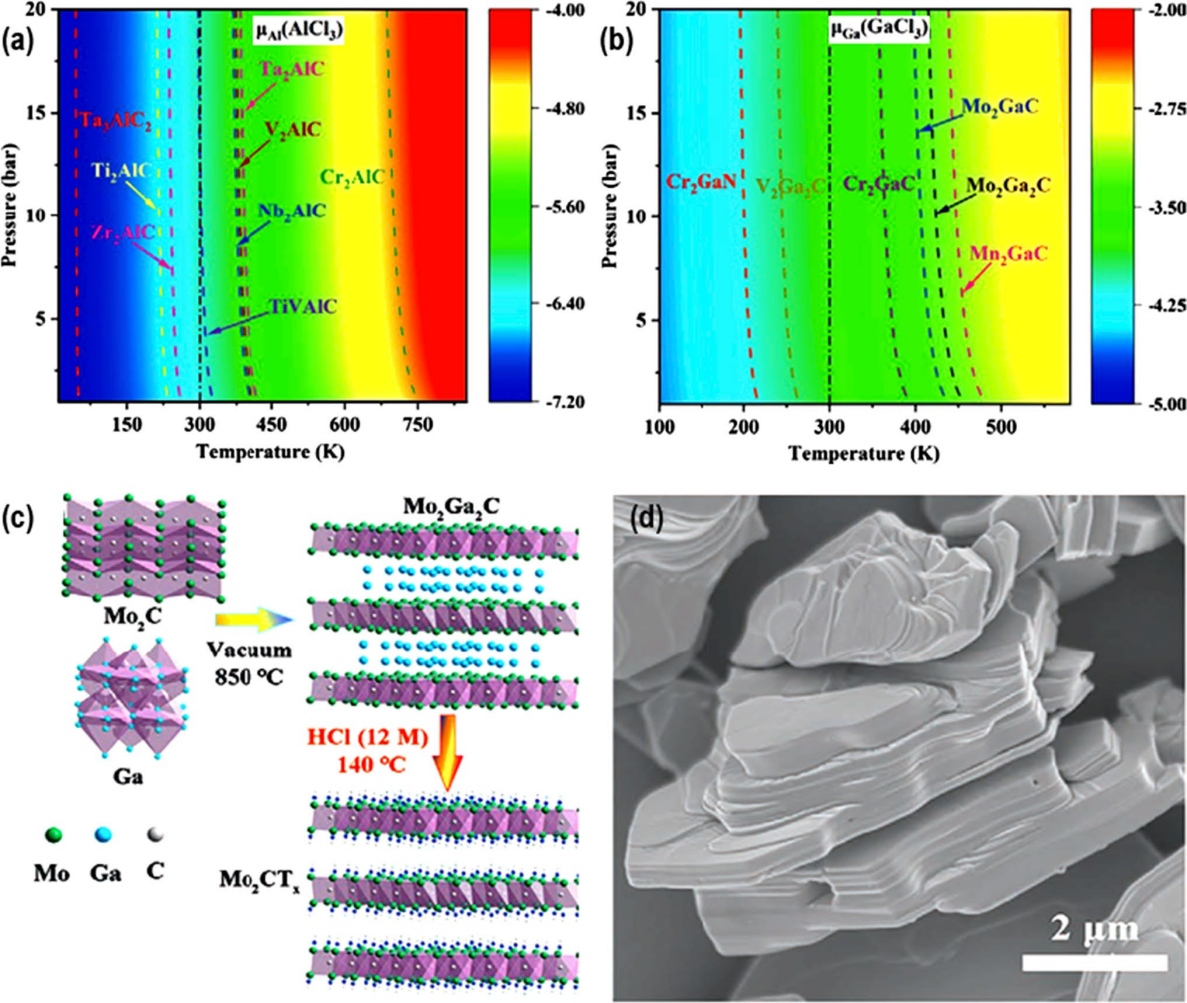
Representation of temperature- and pressure-dependent map of the chemical potential of **a** Al in AlCl_3_ (Al(AlCl_3_)), and **b** Ga in GaCl_3_ (μGa(GaCl_3_)). **c** Preparation strategy for the fluoride-free Mo_2_CT_*x*_, and **d** SEM picture of Mo_2_CT_*x*_ MXene. Adapted with permission [57], Copyright (2021), Wiley–VCH GmbH

Further, using the DFT calculations, Wang et al*.* [57] proposed a strategy (i) to model the practicability of different MAX materials with Al and Ga interlayer elements, (ii) to predict the deciding etching conditions of temperature and pressure (T&P), and (iii) to solve the mystery of selective etching of MAX phases using HCl (Fig. 6a, b). After that, they proposed an experimental method for etching a variety of MAX materials, such as Mo_2_Ga_2_C and Cr_2_AlC, using the HCl-hydrothermal etching method, which is both straightforward and highly tunable (Fig. 6c, d). Compared to Mo_2_CT_*x*_ MXenes, which are synthesized by the HF-etching approach, the as-prepared fluoride-free Mo_2_CT_*x*_ of high quality only exhibits Cl- and O-containing terminations and has a distinct capacitive behavior. Similarly, using hydrothermal etching with HCl, Guo et al*.* [58] effectively synthesized very pure Mo_2_CT_*x*_ MXene from Mo_2_Ga_2_C. Pure Mo_2_CT_*x*_ MXene was obtained in an NH_4_F + HCl solution at a temperature of 140 °C, which is significantly lower than the temperatures required by other etching solutions. The atomic force microscopic (AFM) technique confirmed that the thickness of the as-prepared monolayer Mo_2_CT_*x*_ nanosheet was 1.5–0.3 nm. The Mo_2_CT_*x*_ MXene was shown to be stable in an Ar environment at temperatures up to 500 °C. The Mo_2_CT_*x*_ MXene underwent a series of changes as the temperature rose, culminating in the formation of Mo_2_C/MoC/Mo_3_C_2_ at 700 °C, where it was shown to be stable alongside Mo_2_CT_*x*_ MXene. Further, to check the effect of fluoride on HCl-based hydrothermal etching of V_2_AlC, Wang et al*.* [59], prepared a high-purity V_2_CT_*x*_ MXene. Because of its reduced potential for harm and increased efficiency, this strategy is preferable. It was found through characterization that the produced V_2_CT_*x*_ was of greater purity and had superior electrochemical characteristics as an anode for lithium-ion batteries. By adjusting the reactive conditions of the system, high yields and high purities of V_2_CT_*x*_ produced with various etching systems are possible. However, V_2_CT_*x*_ MXene produced at various etching systems demonstrates widely varying electrochemical performance. The superior performance of V_2_CT_*x*_ produced in a solution of NH_4_F and HCl is attributed to the fact that the interlayer distance between the two atoms has been widened, making active sites for ions more easily accessible, and the resulting lower impedance.

#### Electrochemical etching

An etchant is used in the majority of etching techniques, but it can have some influence on the etching outcomes. When no etchant is utilized, electrochemical etching minimizes the negative consequences of etchant. Exfoliation can also be accomplished electrochemically by increasing the interlayer gap. In contrast to MX binding, which includes a mass transfer from the target material to another material, electrochemical etching involves electron transfer as a component of the surface reaction and is primarily dependent on the chemical activity of the MA bond [60]. By reducing the Van der Waals interactions between layers through ion intercalation, electrochemical methods also create 2D materials such as monolayer 2D flakes [56]. A working electrode for the MAX phase and a counter electrode are submerged in an electrolyte in a typical experiment. Ions are created when a potential is applied, which causes the MAX phase to inflate and create 2D nanosheets [28, 61]. The MAX layers can inflate, separate, and exfoliate when exposed to cathodic or anodic potentials [62]. Two-dimensional materials can undergo cathodic exfoliation in a non-aqueous environment to avoid oxidation and provide high-quality non-oxidized nanoplates. For instance, exfoliation of black phosphorus (BP) via cathodic intercalation of tetra-alkylammonium cation frequently resulted in different layers between 2 and 11 [63, 64]. Anodic etching was used by Yang et al*.* [56] to show an effective fluorine-free etching technique based on Ti_3_AlC_2_ in an aqueous electrolyte (Fig. 5e).

The anode was made of Ti_3_AlC_2_, and the Cl^−^ in the electrolyte quickly corroded the Al and destroyed the Ti–Al bond in the anode. The anode Ti_3_C_2_T_*x*_ (T_*x*_ = OH, O) is an alkaline solution made up of NH_4_Cl and tetramethylammonium hydroxide (TMA-OH). The anode was made of Ti_3_AlC_2_, and the Cl^−^ in the electrolyte quickly corroded the Al and destroyed the Ti–Al bond in the anode. The anode Ti_3_AlC_2_ was quickly totally etched after subsequent embedding of NH_4_OH into the nanosheets and stimulated more etching beneath the surface. As a response, the etching procedure is suggested in Eq. (12):

12 $${\text{Ti}}_{3} {\text{AlC}}_{2} - 3{\text{e}}^{ - } + 3{\text{Cl}}^{ - } \to {\text{ Ti}}_{3} {\text{C}}_{2} + {\text{AlCl}}_{3}$$

MXene was synthesized swiftly using electrochemical etching at room temperature. The investigations also proved that MXene-based all-solid-state SCs had higher capacitance following electrochemical etching than MXene which had been etched using LiF/HCl. As a result, electrochemical exfoliation may be a useful technique for producing MXene nanosheets with the precise size and thickness needed. The low yield of MXene monolayer and the need for harsh reaction conditions for exfoliation, as well as the difficulties in removing electrolytic ions, solvents, and ionic liquids from the finished products, all demand improvement [16]. Other cutting-edge top-down techniques include mechanical, electromagnetic, thermal reduction, ammoniation, algal extraction, and etching [65].

### Bottom-up approaches

In addition to top-down synthetic techniques, bottom-up methods such as chemical vapor deposition (CVD) and physical vapor deposition (PVD) can be used to create bare MXenes. The bottom-up method is a controllable means to generate epitaxial films of MXenes with few layers, in contrast to top-down procedures, which utilized etchant materials to obtain multilayered MXenes.

#### Chemical vapor deposition

The CVD approach offers a suitable alternative to the previously stated ways for producing MXene quickly and with a respectable yield. Ultra-thin MXene materials can be prepared via CVD. By using CVD on a Cu/Mo alloy surface in a CH_4_ environment at 1085 °C, Gogotsi et al*.* [66] created ultra-thin 2D a-Mo_2_C. Mo_2_C crystals' thickness and size were adjusted by changing the experimental circumstances, with growth time and deposition temperature controlling the lateral size and nucleation density of Mo_2_C. This technique can be used to create crystals with a variety of shapes, including triangular, rectangular, hexagonal, octagonal, nonagonal, and dodecagonal ones, which have Mo atoms packaged hexagonally [67, 68]. Furthermore, Jia et al*.* [69] enhanced this technique and published the synthesis of ultra-thin n-Mo_2_C nanosheets utilizing MoO_2_ as a template and source of Mo. By heating a tantalum-copper bi-layer with the proper precursor, Wang et al*.* [70] reported the synthesis of MXenes nanosheets using the CVD technique to produce incredibly thin tantalum carbide, nitride, and boride layers (C_2_H_2_, B powder, and NH_3_). The scratch and oxidation resistance of copper was improved by the strong interface adhesion of these ultrathin materials.

**Fig. 7:**
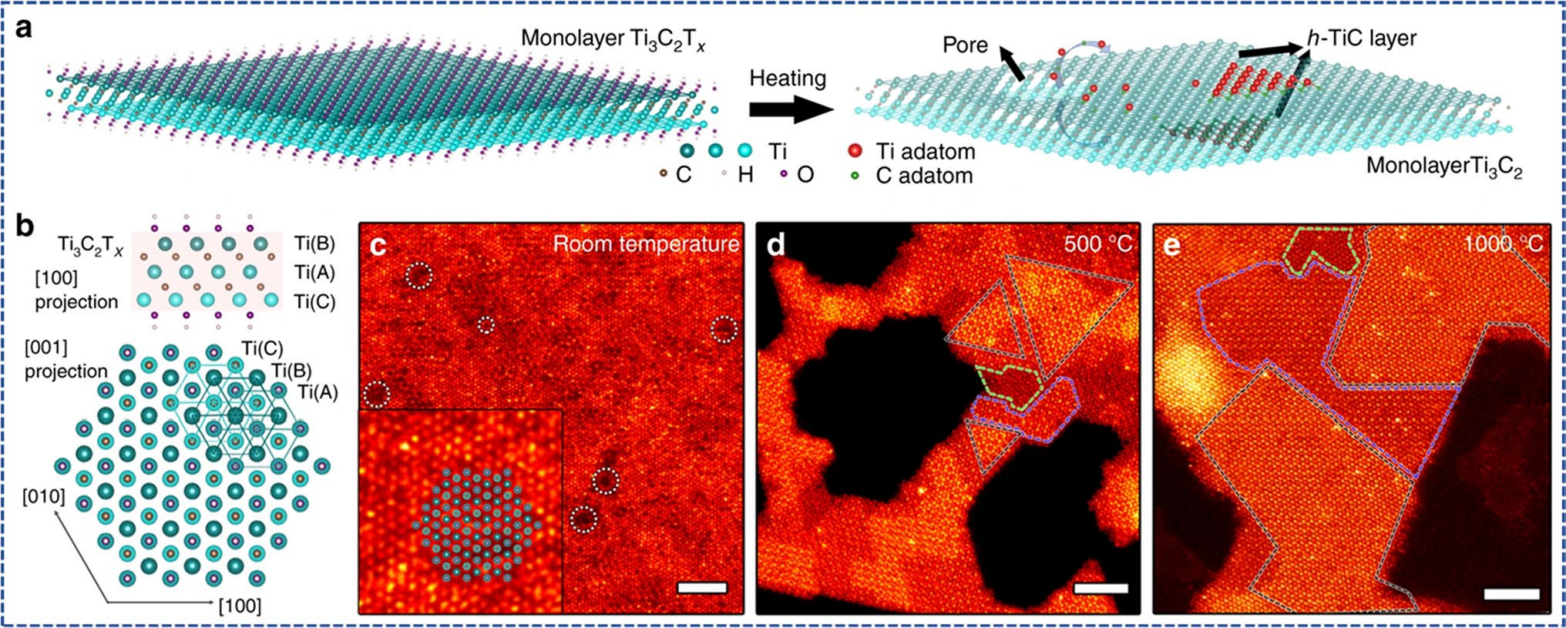
**a** MXene homoepitaxial development. **b** Atomic resolution STEM image from a monolayer Ti_3_C_2_T_*x*_ along the [001] zone axis at ambient temperature. **c** Crystal structure of monolayer Ti_3_C_2_T_*x*_ as seen from [100] and [001] zone axes. **d** A STEM image of a heated monolayer Ti_3_C_2_ flake. **e** A STEM photograph of heated MXene flakes. Adapted from reference [72], Copyright (2018), The Author(s). Published by Springer Nature. This article is an open-access article distributed under the terms and conditions of the Creative Commons Attribution (CC BY) license

The 2D materials created by etching feature surface-ending functional groups, whereas the MXenes created by CVD do not. However, they have unique properties and a stable atomic structure, enabling the investigation of their interior characteristics and their impact on domain boundaries. The intrinsic electrical and optical properties of MXenes may be studied thanks to such bottom-up techniques, which highlights the critical significance of comprehending the MXene function [71]. Using in situ aberration and corrected scanning transmission electron microscopy, Sang et al*.* [72] revealed the homoepitaxial Frank-van der Merwe atomic layer development mechanism of TiC single adlayers formed on surfaces of Ti_3_C_2_ MXene substrates with the substrate serving as the source material as shown in Fig. 7a. At temperatures above 500 °C, hexagonal TiC single adlayers grew on defunctionalized surfaces of Ti_3_C_2_ MXene, activated by thermal exposure and electron-beam irradiation, creating novel 2D materials Ti_4_C_3_ and Ti_5_C_4_ (Fig. 7b–e). Additionally, to increase the cost savings and scalability of MXenes via the CVD approach, high-temperature conditions and pricey sacrificial growth substrates remain barriers.

#### Physical vapor deposition

To create MAX phases, PVD is thought to be a popular technique. Generally speaking, PVD syntheses are performed between 700 and 1000 °C [73]. For example, Cr_2_AlC, Cr_2_GeC, and V_2_GeC may typically couple MAX phases with M-elements of the 5th/6th group at a reasonably low substrate temperature (500 °C), whereas Ti-based MAX phases need a higher temperature [74, 75]. In addition to material selection, efforts to lower the temperature for the substrate include the use of ionized deposition techniques, such as high-power impulse magnetron sputtering and sequential deposition at an appropriate temperature (~ 650 °C for Ti_3_–SiC_2_), which can detach elements and develop MAX phase at a lower temperature. It is typically preferable to repeat sputter deposition under industrial circumstances by controlling particular elements, composites, or compounds from the target composites or compounds. Reactive sputtering with N_2_-gas is typically used to create MAX nitrides [76]. The reactive sputtering method for MAX carbide is somewhat constrained while the process window for producing pure MAX phases is often constricted. However, several studies are showing that the technology has the potential for wider deployment. For the phase MAX synthesis, cathodic arc deposition has a more limited utility than sputtering, for example, Ti_2_AlC creation through a cathodic pulsed arc system from Ti-, Al-, and C- components. The fundamental difference between arc deposition and sputtering is the higher flux ionization degree (almost 100%), which suggests a means to lower temperature [77, 78]. On a sapphire (0001) substrate, Mo_2_C thin films were created by Zhang et al*.* [79] using the plasma-enhanced pulsed laser deposition technique (PLD). The synthesized crystal's structure was discovered to be face-centered cubic with a favored orientation of the < 111 > direction at 700 °C. At a lower temperature, in contrast, the crystal developed an orthorhombic shape. In contrast to CVD, this approach relies heavily on the methane plasma to create Mo_2_C thin films at a very low growth temperature. Pyrolysis and the template method are two further contemporary bottom-up strategies [65].

The top-down synthetic method may produce a variety of MXenes compounds with novel compositions. Additionally, even in the same MXenes and MAX phases, there are numerous probabilities for selectively etching the layer, leading to a variety of surface functional groups, traits, and intended uses. Many bottom-up techniques, including pyrolysis, template method, CVD, and PVD, have been devised in recent years for the production of 2D MXenes. When using this method, superior crystal structure materials are created, as opposed to the MXenes produced using top-down methods. These techniques also enable the synthesis of high-quality 2D MXenes, such as heterostructures, tantalum nitride, tantalum carbide, tungsten carbide, and tantalum nitride. These samples have enormous promise for a wide range of uses, including SCs, batteries, and fuel cells [54]. Although there are still many drawbacks to be solved, such as high temperatures, pricey substrate, harsh environments, less-than-ideal structures and morphologies, and restricted quantities. Therefore, more work is needed to improve the capacity and simplify the preparation processes and growing environments.

### Designing strategies for MXene nanocomposites

Strong van der Waals interactions and hydrogen bonds between neighboring layers during the production process have made it difficult to adopt MXenes for practical use. To address these issues, combining 2D MXenes with other nanomaterials, such as 0D, 1D, 2D, and 3D materials, double-layered hydroxides, conducting polymers, and metal oxide will be a successful strategy [80–84]. Physical or chemical methods could be used to develop design strategies for these nanocomposites.

#### Physical strategy

The physical approach is a straightforward way to create nanocomposites since it allows for the formation of novel products while also avoiding difficult phases in the synthesis process. Ex situ mixing and in situ growth are the two subcategories of the physical synthesis technique [85].

##### In situ growth

**Fig. 8:**
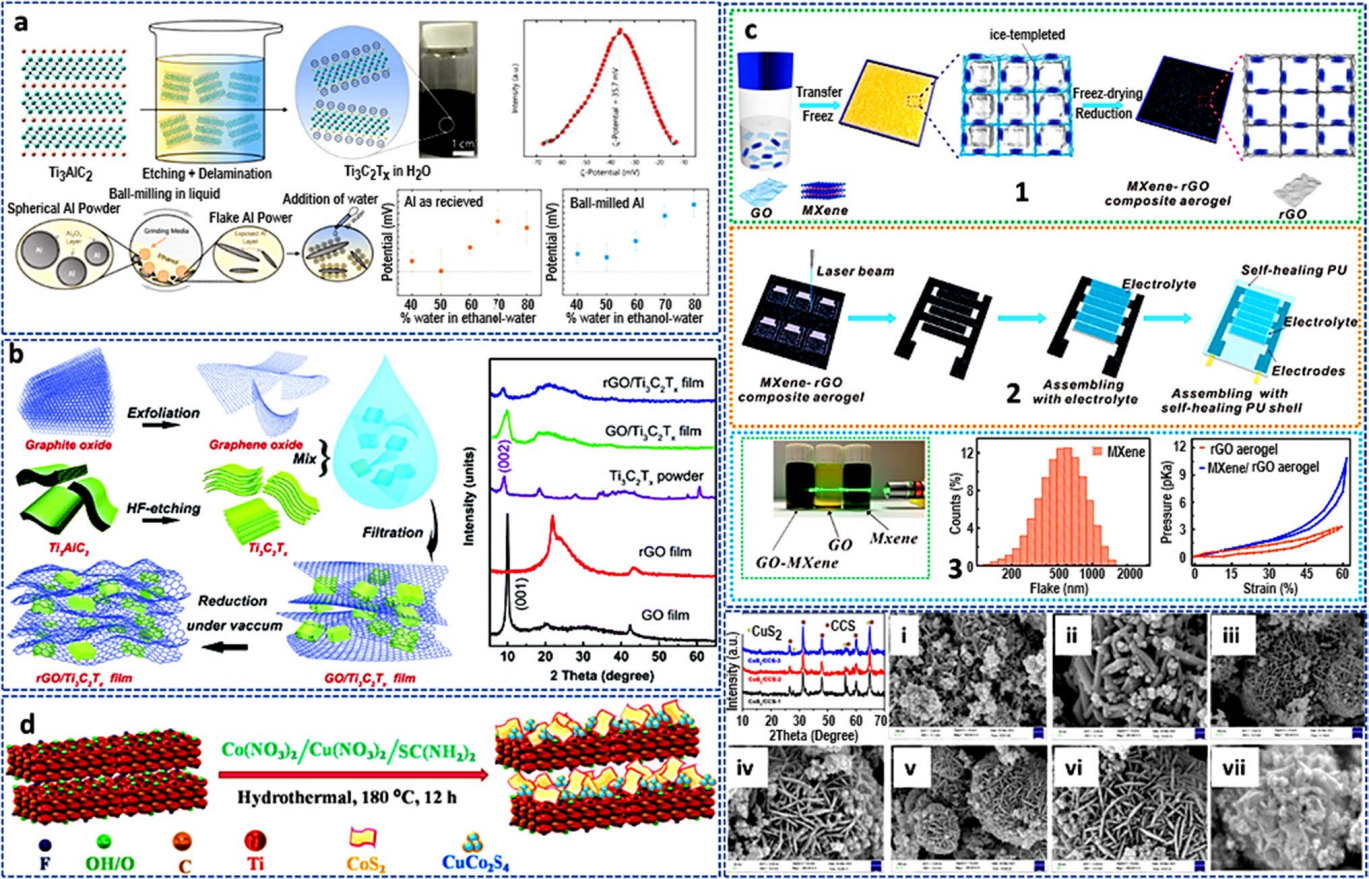
**a** Fabrication and characterization of Ti_3_C_2_T_*x*_ and Al for electrostatic self-assembly. Adapted with permission [89], Copyright (2022), Elsevier. **b** An illustration of how porous rGO/Ti_3_C_2_T_*x*_ films are made, as well as XRD patterns of GO, GO/Ti_3_C_2_T_*x*_, rGO/Ti_3_C_2_T_*x*_ films, rGO, and Ti_3_C_2_T_*x*_ powders. Adapted with permission [93]. Copyright (2017), Royal Society of Chemistry. **c** Schematics of the production of MXene-rGO composite aerogels, along with flake size distributions and pressure–strain curves for the final materials. Adapted with permission [99], Copyright (2018), American Chemical Society. **d** Schematic diagram of the synthesis of the M/CoS_2_/CCS composite. **e** XRD patterns of the CoS_2_/CCS-1, CoS_2_/CCS-2, and CoS_2_/CCS-3 and the FESEM images. Adapted with permission [96], Copyright (2022), Elsevier

In terms of size and form, in situ growth or self-assembly is now the most advanced method for creating nanomaterials. Firm hybrids are produced without the creation of new chemical bonds thanks to the electrostatic attraction between electrolytes with various charges [86]. This method has been successfully used to create a variety of hybrid materials [86–88]. Structures and electrochemical properties are frequently significantly influenced by changing the weight ratio of MXene and its equivalents. To produce single-to-few layer dispersions of Ti_3_C_2_T_*x*_ flakes from 1 to 5 wt.%, Wyatt et al*.* [89] established a self-assembly technique of Ti_3_C_2_T_*x*_ to aluminum that can be adjusted. They also demonstrate how, at concentrations over 5 wt%, the same procedure can be used to incorporate multilayers of Ti_3_C_2_T_*x*_ that have already been pre-stacked or to re-stack multilayers of Ti_3_C_2_T_*x*_ that have been single- to few-flaked (Fig. 8a). The creation of a network of Ti_3_C_2_T_*x*_ in the Al matrix using near-full coverage of Al by Ti_3_C_2_T_*x*_ can be exploited to produce multifunctional structural and/or conductive metal composites. Future additive manufacturing of bulk Ti_3_C_2_T_*x*_–Al metal composites benefited from the ability of this self-assembly technique to produce huge batches of Ti_3_C_2_T_*x*_–Al powder. In the attempt to generate 3D-hydrogel hybrids of MXene and graphene (MGH), Sikdar et al*.* [90] described a wholly room-temperature casting-based method that avoided the potential of oxidation by inducing spontaneous gelation with metallic zinc particles. The MGH was utilized as an SC electrode because of its high mass-specific capacitance (357 F/g at 10 mV/s) and outstanding capacity retention (95.6% after 10,000 charge/discharge cycles). These simple and affordable MXene-graphene hydrogels are a desirable alternative for a variety of applications that call for 3D porous structures.

##### Ex situ mixing

This is a well-known procedure where solutions of dispersed MXene and other nanomaterials are mixed and swirled to enable the interlayers to create a conventional sandwich or hybrid shape. The interlayer gap is increased by these structures, which speeds up ion transport and improves the performance of the nanohybrid. This procedure also involves combining the solutions before alternate layers are deposited [91, 92]. Xu et al*.* [93] discussed the idea of making flexible all-solid-state SCs out of reduced graphene oxide (rGO)/Ti_3_C_2_T_*x*_ film by employing rGO as a binder to link electrochemically active conducting particles. The co-cathode approach was used to combine these SCs with flexible thin-film solar cells to create energy conversion and storage devices ECSDs (Fig. 8b). The reduction at 300 °C while under vacuum was followed by vacuum-assisted filtration of the GO/Ti_3_C_2_T_*x*_ dispersion to create the porous rGO/Ti_3_C_2_T_*x*_ films. By using this method, MXenes were not required to be delaminated, and thick electrodes with adequate electrolyte accessibility could be produced. The composite material, as synthesized, showed a greater specific capacitance than GO, RGO, or MXene.

#### Chemical strategy

Comparatively to MXene nanocomposites made using the physical synthesis approach, chemical strategy is crucial for enhancing their physicochemical and thermomechanical properties. The processes for chemical synthesis include direct thermal treatment, hydrothermal–solvothermal synthesis, and reagent reduction [85].

##### Direct thermal treatment

Without the use of reducing chemicals, thermal or heat treatment techniques can successfully remove active surface functional groups [85]. A MAX (Ti_3_AlC_2_) precursor was used to create a 2D transition metal carbide, MXene (MX–Ti_*x*_C_*x*_T_*x*_), and MX@Pt nanocomposites. The efficient (MXene/Pt) nanocomposite photocatalysts were made using simple water bath sonication and CVD tubular furnace direct thermal annealing. The structural conformation of MX@Pt nanocomposites was also compared to the successfully synthesized MAX into MXene (MX) flakes using XRD data, which showed good crystalline diffracted peaks. MXene nanocomposites with few-layer sheets coated with "Pt" were investigated using surface FESEM morphology [94]. Vacuum freeze-drying (65 °C) under 25 Pa, followed by annealing (300 °C) for 1 h under Ar gas, produced a lightweight GO-based MXene hybrid foam (MX-RGO) [95]. To ascertain the precise physicochemical properties and prospective uses, various foam compositions were created.

##### Hydrothermal–solvothermal synthesis

A common method for creating MXene nanocomposites is hydrothermal–solvothermal synthesis, which is similar to thermal or heat treatment techniques. This process involves placing the reactants in a closed container with a solvent present at a high temperature and pressure for a predetermined amount of time, followed by washing and drying. The reaction must be carried out at medium or low temperatures because extremely high temperatures can damage MXene's structural integrity [85]. The most utilized process of producing nanomaterials or composites is the hydrothermal technique. The nanocrystals formed under conditions of high pressure and temperature have good crystallinity, controlled nanoparticle size, and exceptional dispersibility. Ion or molecule activity is significantly increased in the subcritical state, compared to solid-state reactions, to produce a variety of chemicals [85]. For the design of supercapacitor devices in both symmetric and asymmetric modes, a unique, one-pot hydrothermal synthesis of Ti_3_C_2_T_*x*_ (MXene)/CoS_2_/CuCo_2_S_4_ nanohybrid with various reactive equivalents was demonstrated (Fig. 8d). A morphological investigation demonstrated the successful coating of CuCo_2_S_4_ particle- and sheetlike CoS_2_ on Ti_3_C_2_T_*x*_ nanosheets (Fig. 8e). The electrochemical efficiency was improved by varying the MXene to CoS_2_/CuCo_2_S_4_ ratio. This suggested MXene-based hybrid nanocomposite electrode has tremendous potential for use in future energy technologies, thanks to its exceptional cycle life and high electrochemical energy storage efficiency [96]. MXene/SnTe nanocomposites were produced in situ by Jiang et al*.* [97] using a simple solvothermal technique. Comprehensive characterization results showed that the addition of 2D MXene to the SnTe matrix can suppress Sn vacancies to produce a lower carrier concentration and induce abundant MXene/SnTe interfaces, which simultaneously enhanced the electrical and thermal transport properties. Their work investigated a novel approach to integrate 2D MXene into SnTe-based materials with enhanced thermoelectric properties, which can open up new avenues for developing and producing high-performance thermoelectric materials.

##### Reagent reduction

During the production of MXene nanocomposite, a reagent or chemical is given to the reaction medium to react with a precursor by a procedure known as chemical reduction [85]. Ti–O–C covalent connections were formed to create rGO/MXene sheets, which are extremely strong MGO sheets (MXene and GO). The created rGO/MXene sheets were cross-linked by 1-aminopyrene-disuccinimidyl to minimize the voids within the GO layers and improve the symmetry of the arrangement of GO platelets, which gave the material super-toughness [98]. To create GO/MXene sheets, the dispersed GO and MXene media were sonicated for 10 min.

After that, the mixture was mixed for 6 h, vacuum-assisted filtered, and dried at 60 °C for 12 h. After multiple washes with dimethylformamide (DMF) and ethanol, the GO/MXene sheets were reduced with HI and then added with 1-aminopyrine-disuccinimidyl to produce the desired product. By freeze-drying and using reducing agents, a highly self-healable 3D rGO/MXene (Ti_3_C_2_T_*x*_) composite aerogel was created [99]. The performance of the composite was first optimized using several GO/MXene solutions. The aerogel was created by freeze-drying the solutions. The aerogels were then added to a solution of acetic acid and hydrogen iodide at 60 °C for 3 h, after which washing and freeze-drying were performed. The rGO/MXene (Ti_3_C_2_T_*x*_) electrode was then made utilizing a laser cutting technique as described in Fig. 8c. A 3D self-healable rGO/MXene (Ti_3_C_2_T_*x*_) was made from this material, which was once treated with a carboxylated polyurethane shell. The finished product was used in a supercapacitor.

## Properties of MXenes

MXenes exhibit a unique blend of traits, including excellent electrical and mechanical performance. MXenes stand out from other 2D materials, such as graphene, due to their hydrophilic nature combined with improved thermal conductivities [100]. Through linkages between morphology and geometric structure, surface functioning, and composition, the associated features and expulsive performances can be changed [101, 102].

### Structure of MXenes

**Fig. 9:**
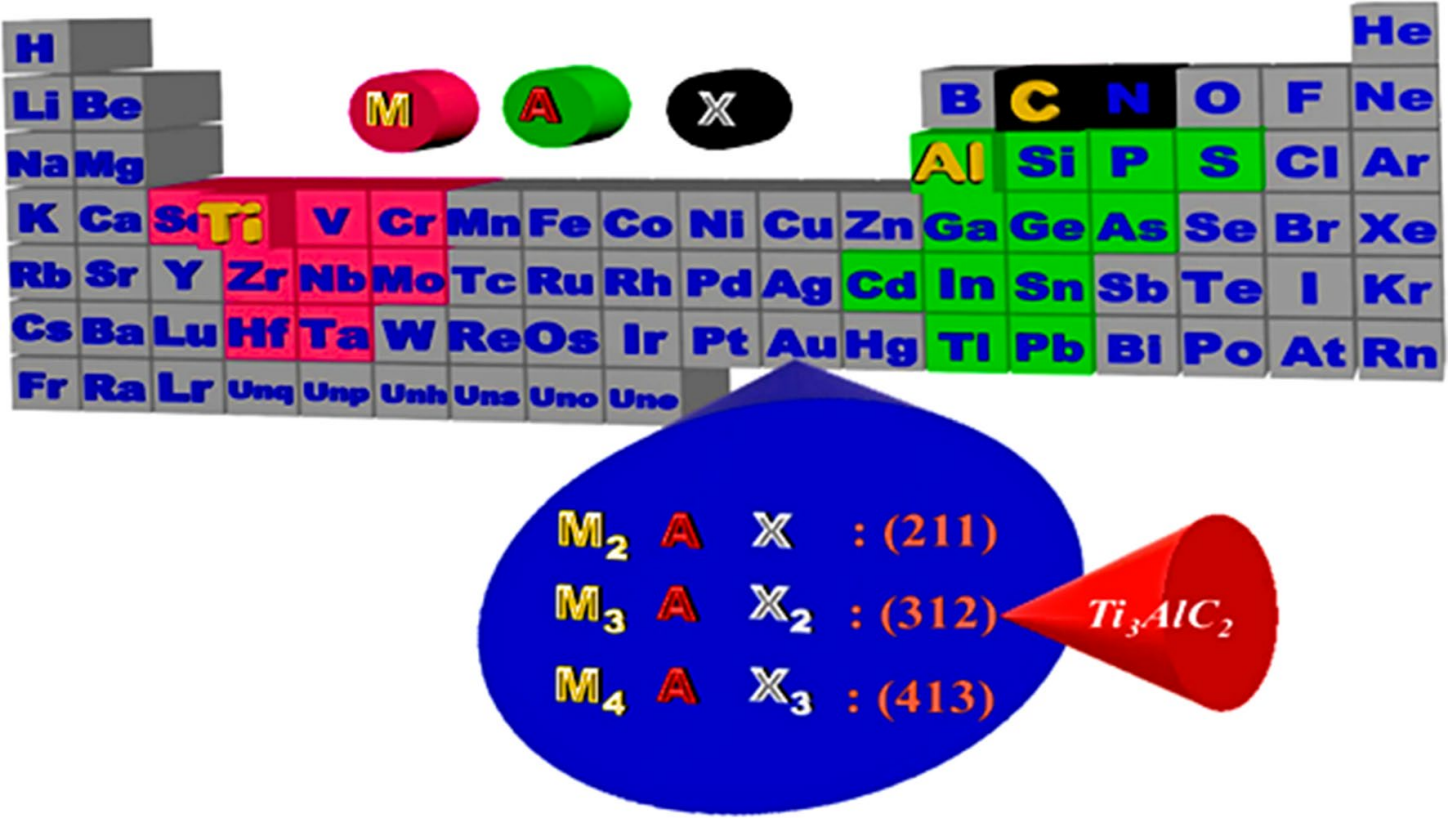
A representation of the location of a MAX phase's constituent elements in the periodic table. Adapted with permission [107], Copyright (2021), Elsevier

In the precursor material MAX phase, group A elements (Fig. 9) are selectively etched to produce MXenes. The more chemically reactive Group A elements can be removed by etching because the MX bonds are stronger than the MA bonds, while the layered structure of M_*n*+1_X_*n*_ is retained [103, 104]. MXenes have the generic formula M_*n*+1_X_*n*_T_*x*_ (*n* = 1, 2, 3), where M represents transition metals like Ti, Mo, Cr, Nb, and V, X is carbon or nitrogen, and T_*x*_ stands for different surface functional groups like –OH, –O, and –F [105]. MXenes are made up of many layers of hexagonal crystal cells. Wherever the X atoms fill the octahedral interstitial locations, the M atoms are packed hexagonally [106].

With a change in the amount of *n* in the general formula, the arrangement pattern of MXene atoms will alter. The hexagonal dense packing of M_2_X MXenes contrasts with the cubic dense packing of M_3_X_2_ and M_4_X_3_ MXenes. The M layer atoms in MXenes form a total of 6 chemical connections with the chemical groups linked to the surface and the adjacent X layer atoms since the coordination number of transition metal ions is typically 6, which gives MXenes their surface group T_*x*_. The applications of MXene-based flexible sensors and supercapacitors are greatly impacted by these highly adaptable surface groups, which are one of the qualities that set MXenes apart from 2D materials like graphene and transition metal sulfides. Surface groups, for instance, may impact on MXene's capability for material fusion [108] as well as the adsorption potential of particular detection molecules [109].

### Electronic properties

**Fig. 10:**
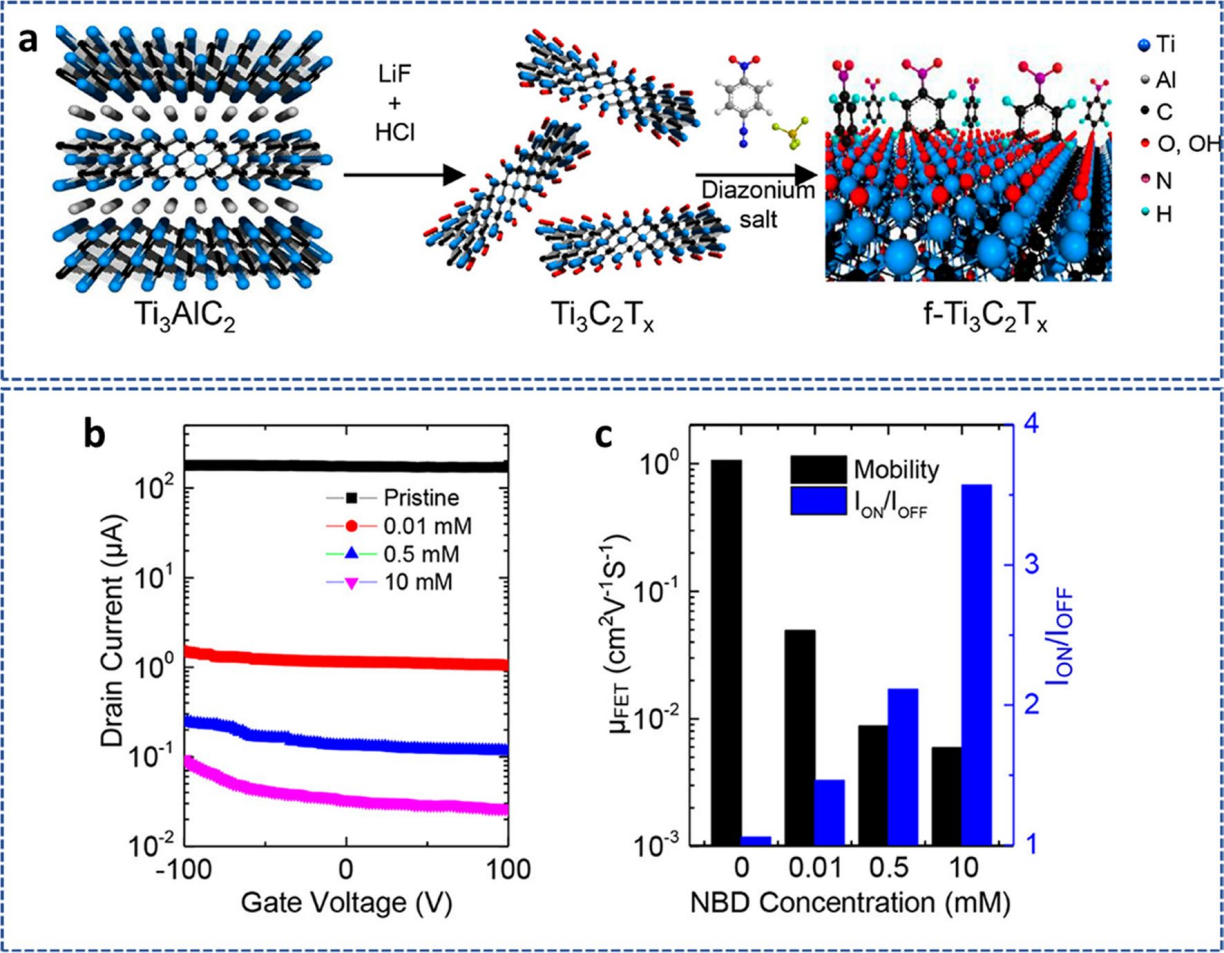
**a** A schematic showing the synthesis of Ti_3_C_2_T_*x*_ and its subsequent diazonium functionalization. **b** Transfer curves for 10 mM, 0.01 mM, and 0.5 mM and pristine Ti_3_C_2_T_*x*_. **c** The Ion/Ioff of f-Ti_3_C_2_T_*x*_ and field mobility μFET. Adapted with permission [111], Copyright (2021), American Chemical Society

The electronic property of MXene is a significant aspect that is of primary importance. The surface groups (–OH, –F, –O) on MXenes can be changed by adjusting their functional groups, material balance, or creation of solid solutions, as well as by manipulating the reaction circumstances [110]. For instance, the conductivity and mobility of single-layer Ti_3_C_2_T_*x*_ MXene that was chemically modified using 4-nitrobenzene-diazonium tetrafluoroborate salts decreased as the concentration of the 4-nitrophenyl group was grafted onto the surface of MXene (Fig. 10a–c) [111]. The electrical characteristics of MXenes, including work function, conductivity, and mobility, can also be altered by manipulating surface groups of MXenes by substitution and elimination processes in the molten inorganic salt [112]. The procedure used to create MXenes also has some influence on electrical characteristics in addition to the influencing elements mentioned above. Better conductivity for the same type of MXene is frequently obtained by synthesizing it under softer etching conditions. This is because only a little etching can effectively preserve MXene's structure, and the produced nanosheets are both big and relatively free of flaws.

The high electron density of MXenes around the Fermi level caused by the d-orbital electrons of the transition metal layer elements and the interior metal carbide layer's ability to effectively transport electrons are what cause their high conductivity [113, 114]. Additionally, alterations in the surface groups and transition metal layer components have a significant impact on the electrical characteristics of MXenes. For instance, Ti can be switched out for Mo in the outer transition metal-M layer of M_3_C_2_ and M_4_C_3_ MXenes to vary the material's behavior in terms of how electrons are transported from a metal to a semiconductor. Figure 10a demonstrates that at any temperature, the replacement of Mo-containing MXene has a higher resistivity than the corresponding Ti-containing MXene. However, the Mo-containing MXene with the surface group –O exhibited the metal electron transport behavior, unlike the Mo-containing MXenes with the surface groups –OH or –F [115]. Furthermore, the electrical conductivities connected to the pressed films of MXenes coincided with those of multilayer graphene and were greater than those in nanocarbon tubes. It was evident that functional molecules might hinder resistivity growth by increasing it with different layers. As a result, the conductivities of simulated functions have maximal values compared to observations from experiments [116]. The electrical conductivities of Ti_3_C_2_T_*x*_ varied from 850 to 9870 S/m because of differences in the following factors: surface functionality; defect concentration; d-spacing between MXenes particles; delamination yield; and lateral dimensions brought about by the etching procedure [117]. Generally, increased lateral expansion and shorter etching times combined produce MXenes with fewer flaws and greater levels of electrical conductivity [118]. Furthermore, environmental wetness may affect their electrical conductivities, which would encourage them to be used in sensing applications [119].

### Mechanical properties

**Fig. 11:**
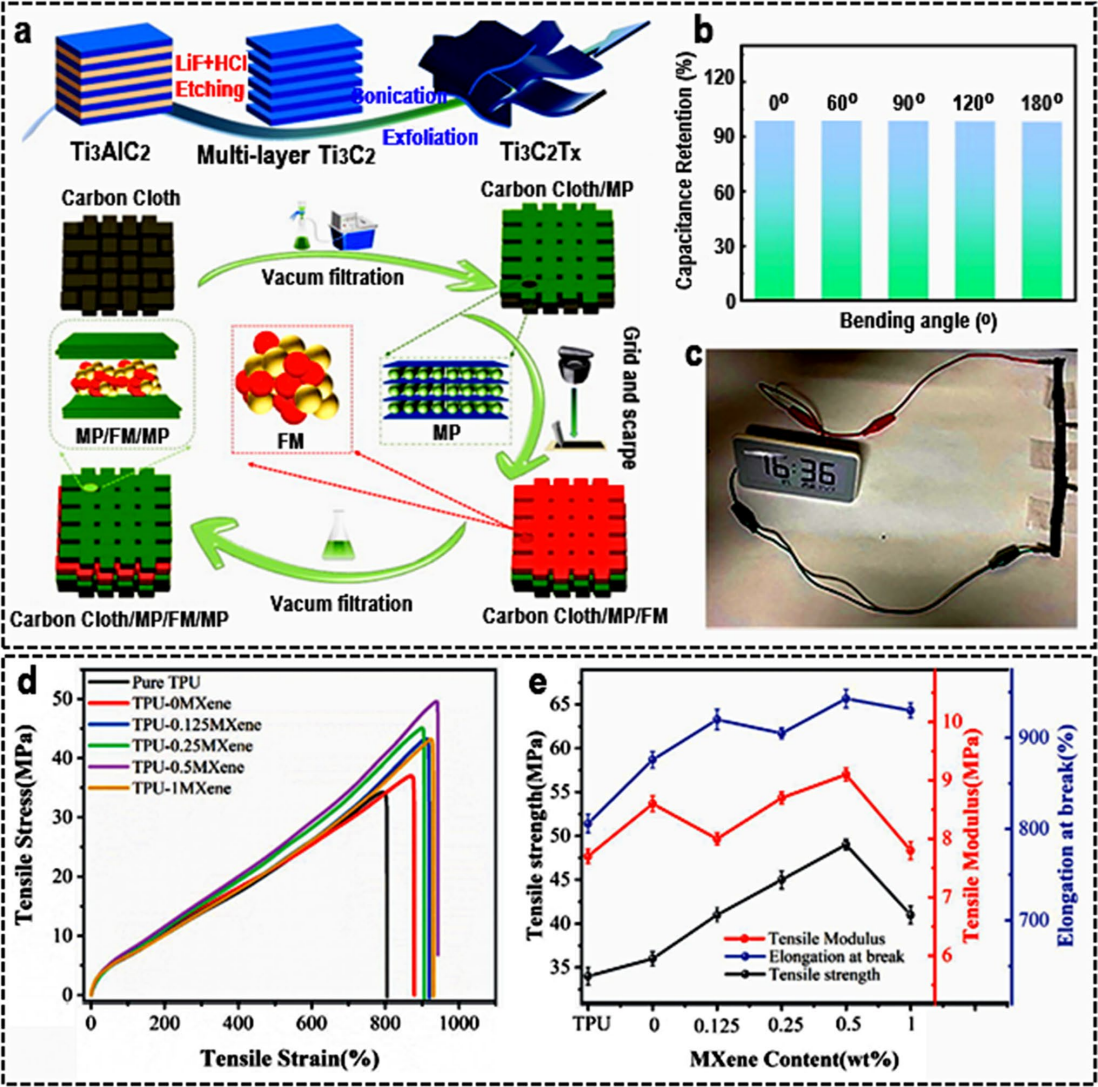
**a** An illustration showing how to make MP/FM/MP and MXene films on carbon cloth. **b** Capacitance retention at various bending angles. **c** Electronic meter driven by three supercapacitors. Adapted with permission [122], Copyright (2022), Elsevier. Mechanical features of pure TPU and MXene/TPU hybrid with various loading of Ti_3_C_2_T_*x*_ MXene; **d** stress–strain curves, and **e** tensile strength, tensile modulus, and elongation at break. Adapted with permission [123]. Copyright (2022), Wiley and Sons

Surface terminations have a significant impact on the mechanical properties of MXenes. The O-end MXenes are anticipated to be quite rigid, but MXenes ended by other groups (F and OH) display lesser elastic stiffness and toughness in comparison with their O-end counterparts [120]. Its distinctive lattice coefficients, which include many terminations, can be likened to those of MXenes: the lattice limits of O-ended MXenes are often greater than those of F or OH-removed MXenes [121]. Particularly, when compared to straightforward MXenes, surface-functionalized MXenes offer greater flexibility and adaptability. By creating a sandwich structure, Li et al*.* [122] for the first time added a-Fe_2_O_3_/MnO_2_ (FM) to MP. The MXene surface's numerous active sites and favorable hydrophilic characteristics allowed FM to interact with the MP (Fig. 11a). The accumulation and loss of FM were minimized by complexing with MP. Additionally, the impact of increased loading on MP was superior mechanical qualities and enhanced flexibility. To show that it can be utilized as a flexible energy device, they tested its flexibility. Flexibility tests were performed on the final product between 0 and 180 degrees [122]. The results, as presented in Fig. 11b, made it abundantly evident that even at a 180° bending angle the single capacitor's capacitance retention can reach 98.2% and that its CV curve virtually overlaps that without bending. As shown in Fig. 11c, the electronic meter was powered by three capacitors. In another study, Qingsen et al*.* [123] used Ti_3_C_2_T_*x*_ MXene nanosheets that had been pretreated with polyethylene glycol to create a new nanocomposite by melting them together with thermoplastic polyurethane (TPU). Tensile strength and elongation at break were observed to rise by 41.2% and 15.4%, respectively, at MXene loading values of 0.5 wt.% (Fig. 11d, e). TPU's crystalline speed is increased slightly by MXene, and the movement of tiny gas molecules is effectively slowed down when temperatures are high. Low-frequency properties of TPU nanocomposites as storage modulus, loss modulus, and complicated viscosity improve as MXene concentration rises. The findings show that TPU nanocomposites can benefit from MXene nanoparticles in mechanical, thermal, and rheological aspects. Within this framework, to enjoy the mechanical features of MXenes, the concerned scientific community is focusing on using the conducting polymers with the MXenes to improve their performance in multifunctional applications. Table 1 summarizes the main parameters in the mechanical properties of MXene-based materials.

**Table 1:** Mechanical properties of MXene/polymer membranes

MXene/polymer	Method	Wt. (%)	*t* (μm)	Tensile strength (MPa)	Strain (%)	Young’s mod. (GPa)	Refs.
Ti_3_C_2_/polyvinyl alcohol	Vacuum-assisted filtration	40	12.0	91 ± 10	4.0 ± 0.5	3.7 ± 0.02	[124]
Ti_3_C_2_/Chitosan	Drop casting	2	70 − 120	48.4	15.2	–	[125]
Ti_3_C_2_/Sulfonated poly(ether ether ketone)	Drop casting	2	70 − 100	41.3	76.2	–	[125]
Ti_3_C_2_/PEDOT:PSS	Vacuum-assisted filtration	75	15.2	30.18	1.51	–	[126]
Ti_3_C_2_/Thermoplastic polyurethane	Hot press	0.5	1000 ± 300	55.9 ± 4.6	605 ± 22	–	[127]
Ti_3_C_2_/ polybenzimidazole	Drop casting	5	155 ± 6	53	2.7 ± 0.2	–	[128]
Ti_3_C_2_/Thermoplastic polyurethane	Hot press	0.5	1000 ± 300	50.8 ± 1.1	599 ± 45	–	[127]

### Electrochemical properties

Based on Bader charge analysis, the M element in the MXene is in a substantially lower oxidation state than its corresponding oxides (the most stable thermodynamic class). MXene can consequently oxidize as a result. Additionally, termination groups have a significant impact on the oxidation state of the MXene's M element, and this state can be controlled via moderate redox oxidization, in which MXene acts as a reducing agent and noble metal ions act as oxidants [129]. In contrast to total oxidation into oxides, moderate oxidation also preserves structural integrity and allows for the production of noble metal nanoparticles. The equally distributed noble metal nanoparticles in MXene and the appealing enhanced Raman scattering at the surface are striking [130]. Due to the abundance of redox sites, MXenes and Ti_3_C_2_T_*x*_ MXenes in particular, have strong electrochemical properties. Due to the hydronation of the terminal group containing oxygen, the Ti oxidation state continually changes. In other words, the relationship between the change in potential and the change in the Ti oxidation state is almost linear. The Ti_3_C_2_T_*x*_ has various capacitance characteristics that depend on the size of the electrolyte's cations, surface terminations, morphology, interlayer spacing, etc. The electrostatic pull between opposing charges is produced when electrolytic cations intercalate between the Ti_3_C_2_T_*x*_ interlayers. Therefore, if the size of the cations is smaller than the interlayer spacing, they will intercalate through the interlayers. Due to the rapid redox reaction, this will result in pseudocapacitance behavior. Large cations, on the other hand, will not fit through the interlayers and will repel one another electrostatically, preserving excellent cycle stability. In order to produce the electric double-layer capacitance, the cations will adsorb at the surface [131]. Due to this, MXene has a remarkable capacity for storage in acidic solutions. The following (Eq. 13) is a representation of the electrochemical reaction [132]:

13 $${\text{Ti}}_{3} {\text{C}}_{2} {\text{O}}_{x} \left( {{\text{OH}}} \right)_{y} {\text{F}}_{z} + \delta {\text{H}}^{ + } + \delta {\text{e}}^{ - } \leftrightarrow {\text{Ti}}_{3} {\text{C}}_{2} {\text{O}}_{x - \delta } \left( {{\text{OH}}} \right)_{y} + \delta {\text{F}}_{z}$$

**Fig. 12:**
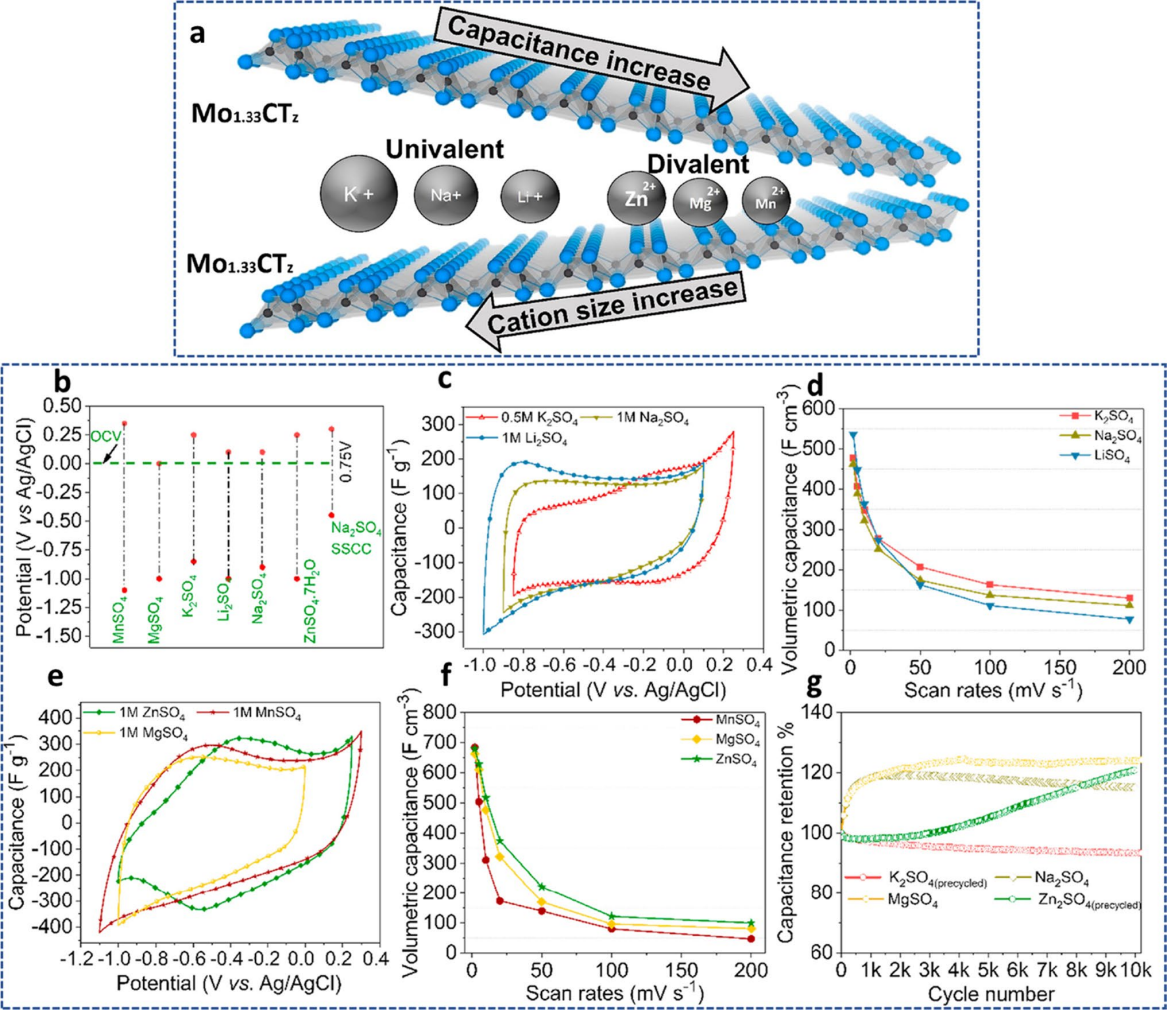
**a** A schematic representation of the Mo_1.33_CT_z_ MXene. **b** Operating potential window; SSCC stands for the stainless-steel current collector; **c** and **d** CV profiles at a scan rate of 2 mV/s. **e** and **f** Volumetric capacitance variation with scan rate. **g** The capacitance retention at a current density of 10 A/g in electrolytes with 1 M NaSO_4_, 1 M ZnSO_4_, 1 M MgSO_4_, and 0.5 M K_2_SO_4_. Adapted with permission [135], Copyright (2022) by the authors. Published by Elsevier B.V. This article is an open-access article distributed under the terms and conditions of the Creative Commons Attribution (CC BY) license

As determined by nuclear magnetic resonance NMR analysis, ${\text{Ti}}_{3} {\text{C}}_{2} {\text{O}}_{x - \delta } \left( {{\text{OH}}} \right)_{y + \delta } {\text{F}}_{z}$ was the predicted surface chemistry for MXene produced using the in situ HF technique, giving it a maximum theoretical capacity of 615 C/g. Though MXenes have only been reported to have an experimental capability of about 135 C/g. Gogotsi et al*.* [132] performed numerous tests on a 90 nm thick MXene film to investigate this discrepancy between theoretical and actual results. They concluded that glassy carbon, as opposed to platinum or gold, might significantly increase the capacity of the MXenes. The limited usage of active sites and incomplete redox reactions constrained by the narrow potential window were potential causes. Additionally, the Pt or Au could restrict the process by catalyzing water splitting, whereas glassy carbon has outstanding overpotential for oxygen evolution reaction (OER) with a wide potential window (> 1.0 V), allowing the inherent capacity of different materials towards OER, making glassy carbon as the foremost current collectors in energy storage as well as water splitting applications. Thus, a 90 nm thick MXene sheet with a high capacitance of 450 F/g and outstanding electrochemical performance was reported by Gogotsi and colleagues. Surface modification or heteroatom doping is another method for increasing the capacitance of MXenes. As an illustration, by combining solvothermal and ex situ nitrogen doping using urea as a nitrogen source, Que et al*.* [133] were able to manufacture nitrogen-doped MXene (NTi_3_C_2_). The highest value of ultrahigh capacitance ever recorded up to this point was found in the synthesized N-Ti_3_C_2_ electrode, which measured 927 F/g. Another illustration is the electrochemical efficiency of the Ti_3_C_2_T_*x*_ anode material for LIBs, which Li et al*.* [134] described. For the Ti_3_C_2_T_*x*_ anode at 0.1C, capacity values of 450 and 250 mAh/g, respectively, were obtained during the initial discharge and charge. Additionally, the produced Ti_3_C_2_T_*x*_ demonstrated good cycling stability; after 1600 cycles at 5C, a capacity number of 119 mAh/g was attained. It has been discovered that the preparation variables, functional groups, thermal treatments, and chemical oxidation are the primary parameters impacting the electrochemical characteristics of MXene anodes, many of them strongly interconnected. El-Ghazaly et al*.* [135] examined the electrochemical behavior of i-MXene (Mo_1.33_CT_z_), a vacancy-containing material (Fig. 12a), in sulfate-based aqueous electrolytes with univalent (Li^+^, Na^+^, or K^+^) or divalent (Mg^2+^, Mn^2+^, or Zn^2+^) cations. The findings demonstrate that the Mo_1.33_CT_z_ MXene electrodes were not degraded in these sulfate electrolytes when operated in a potential window greater than 1 V. The volumetric capacitance of the Mo_1.33_CT_z_ MXene electrodes, as measured in 1.0 M MnSO_4_ solution reached up to 677 F/cm^3^. After 5000 cycles of charging and discharging, the asymmetric devices still held 97% of their initial capacitance. The results of the analysis are displayed in Fig. 12b–g. This suggests that using intercalating cations can improve Mo_1.33_CT_z_ MXene’s electrochemical performance.

Using Zn as the anode and Ti_3_C_2_T_*x*_ as the cathode, Li et al*.* [136] investigated the charge storage mechanism of the Ti_3_C_2_T_*x*_-based-Zn-ion hybrid micro-SC. To create the highly effective electrochemical SC, the researchers discovered that during the discharge process, Zn changed to Zn^2+^ and transported from the anode to the Ti_3_C_2_T_*x*_ MXene cathode, intercalating the layers of MXene. The Zn^2+^ ion intercalation into MXene layers was revealed by the SEM examination, which also revealed the dispersion of Ti, C, and Zn components in the cathode [136]. Generally speaking, the electrochemical performance of MXene-based supercapacitors varies depending on the synthesis techniques, MXene precursors, dimension and architecture of MXene, electrolyte, electrode architecture, and their dispersion as fillers in different composites.

## MXenes for electrochemical energy storage devices

### Types of electrochemical energy devices

The development of clean, effective, and sustainable energy conversion and storage technologies has become one of the imperative strategies for the global science and technology community as a result of the rise in energy demand. Electrochemical technologies, including fuel cells, batteries, and supercapacitors, have been acknowledged as being among the most significant energy conversion and storage techniques. Due to their many benefits, including quick charging, long charge/discharge cycles, and wide working temperature ranges, supercapacitors have several uses in electronic devices, airplanes, smart grids, hybrid/electric vehicles, and other industries. Batteries have also been employed in several applications including stationary and mobile systems [6]. However, there are still several issues with electrochemical energy systems, including a low energy density for SCs and high costs of manufacturing, and low power density for batteries. Supercapacitors and batteries are the two main EES technologies that will be discussed; in particular, their basic principles, mode of operations, and applications based on MXene materials will be emphasized.

#### Supercapacitors

Electrochemical devices also known as supercapacitors (SCs) are used to store energy by collecting charge or through faradic reactions at the interface of the electrode and electrolyte. Due to the quick adsorption–desorption of charged ions, ultrahigh power densities are produced during charge storage in an SC. Additionally, SCs have a longer lifespan than batteries and are linked to relatively low specific energies. Based on the electrode materials employed in electrode manufacturing, SCs may be divided into three types: pseudocapacitor, electrochemical double-layer capacitor (EDLC), and hybrid capacitor.

The arrangement of charges/ions at the electrode/electrolyte interfaces causes a double layer to develop on its own when a conducting electrode is put into a solution of an ion-conductive electrolyte. This is the basic operation of the EDLC. The EDLC, in which charges/ions are physically held via electrostatic charge adsorption at the electrode/electrolyte interface, is the most extensively used SC. The fact that there is no charge transfer at the electrode and electrolyte interfaces is one of the most distinctive features of EDLCs. EDLCs are mostly carbon-based materials.

**Fig. 13:**
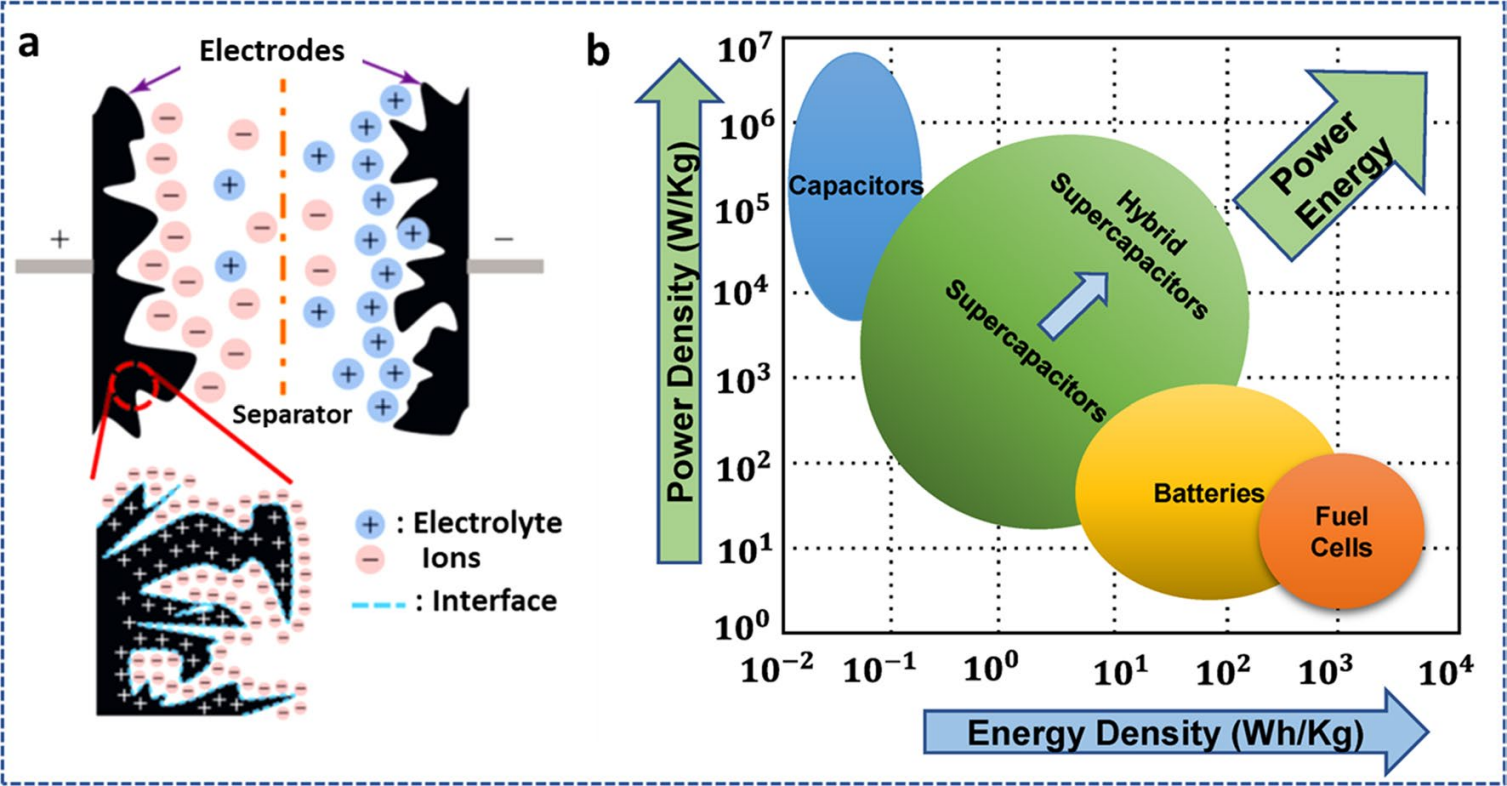
**a** Schematic representation of EDLC operation. Adapted with permission [8], Copyright (2015), John Wiley and Sons. **b** Comparison of energy and power density of various types of capacitors and devices. Adapted from reference [137], Copyright (2021) by the authors. Licensee MDPI, Basel, Switzerland. This article is an open-access article distributed under the terms and conditions of the Creative Commons Attribution (CC BY) license

The specific capacitance and performance of the EDLC device are significantly influenced by the accessible surface area of the various electroactive materials and the surface properties of the carbonaceous materials. Upon the application of a voltage, a double layer is created between the electrode/electrolyte interface as shown in Fig. 13a. Figure 13b compares the energy and power density of various types of capacitors and devices. This causes the adsorption and desorption of the ions. Pseudocapacitors, in contrast to EDLCs, store charge by Faradaic processes, which involve fast and reversible redox reactions at/near the surface of the electroactive materials. This mechanism is connected to the valence state of the electrode material changing primarily as a result of electron/ionic transfer. The first electrode material to display pseudocapacitive properties was RuO_2_. Despite undergoing a Faradaic reaction, the charge storage that results from a charge transfer reaction on a thin RuO_2_ film typically has a rectangular shape, which is a property of various capacitive materials. Electrode materials with capacitive electrochemical properties that store charge by charge transfer of Faradaic processes across the double layer of the device are known as pseudocapacitors.

EDLCs have lower energy densities but long durability, very high specific power, and function well in both aqueous and non-aqueous electrolytes. On the other hand, pseudocapacitors have higher specific energies and specific capacitance, but due to their irreversible redox processes, they have a narrow operating potential window and poor cycling stability. To take advantage of both the EDLC and pseudocapacitive materials, a hybrid capacitor—a single capacitor cell that uses both material features—has been developed. Both capacitive and redox electroactive materials are impregnated in a thin film of polymeric electrolyte where charge storage happens via reversible redox mechanisms. The performance of the hybrid cell as a whole is influenced by both electrochemical and electrostatic causes. The hybrid supercapacitor's capacity for charge storage is proven over a wide range of potentials, providing more specific power and energy without compromising cycle stability.

#### Batteries

The primary research goals in energy storage systems continue to be the creation of positive and negative electrode materials with high capacity, great cycle stability, low cost, and high efficiency. Several materials have been employed as electrode materials for various battery systems because of their outstanding qualities such as high conductivity, solid structural stability, and changeable shape. For a variety of uses, batteries including lead–acid, Ag–Zn, Ni–Cd, Na–S, etc., have been created. Battery cycle lifetimes are typically around 1200 cycles. Because they are robust even in small quantities and have high energy densities, various battery systems have attracted attention. In some works, electroactive organic compounds are included in electrodes made for Li-ion batteries [138]. When added, they offer substitute ions, such as Na^+^, Mg^2+^, and Ca^2+^. Some metal ions, enhance the functionality of battery systems. For instance, prior studies have shown that organic batteries with potassium ions introduced function better [139]. Batteries have also been made with graphene and other carbon-based materials. Through electrochemical species intercalation, interconnected networks, wide surfaces, and large pore sizes, energy is stored in batteries [140].

**Fig. 14:**
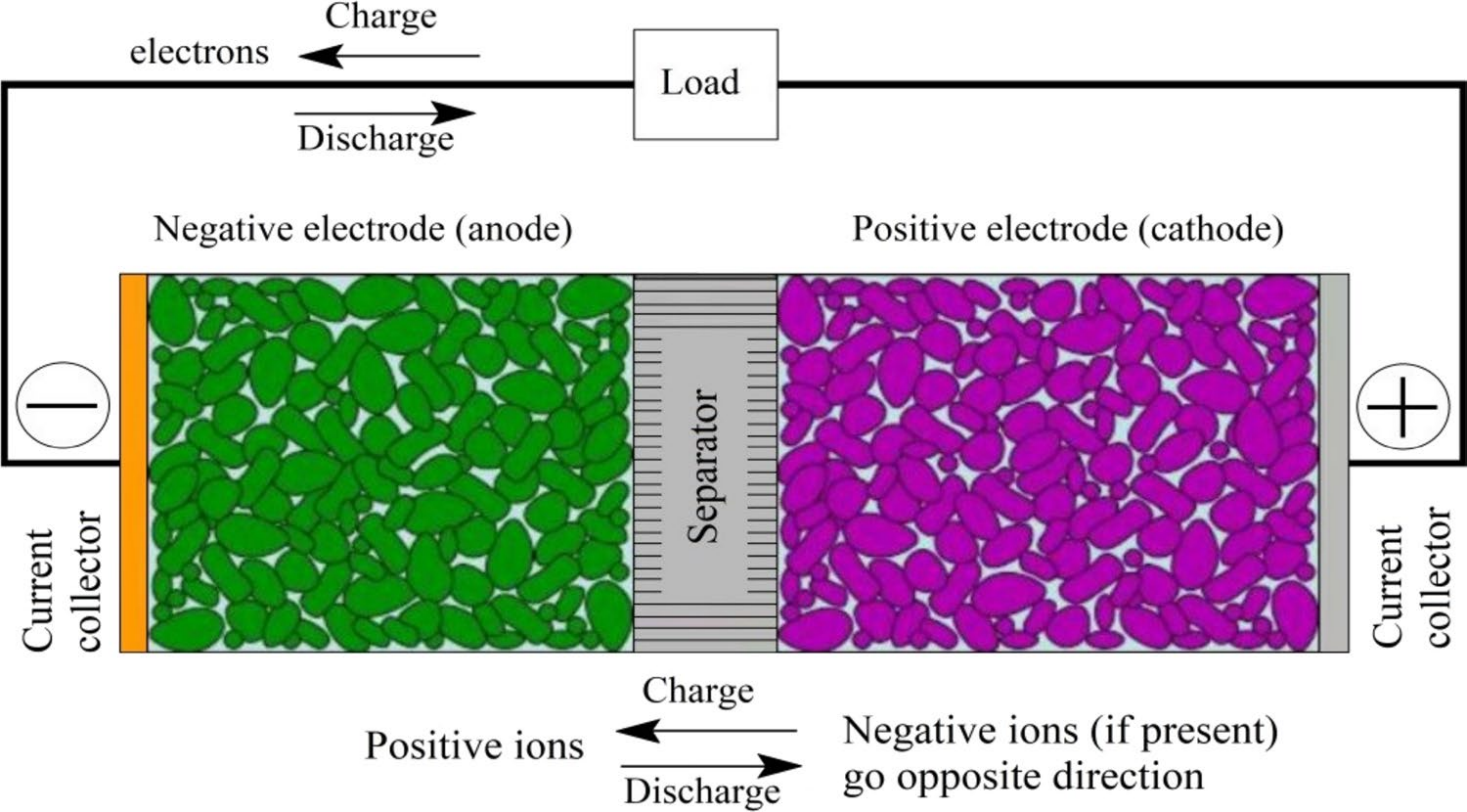
Schematic structure of the interior of a Li-ion battery. Adapted from reference [142], Copyright (2020) by the authors. Licensee MDPI, Basel, Switzerland. This article is an open-access article distributed under the terms and conditions of the Creative Commons Attribution (CC BY) license

According to the contact of electrons on the metallic electrodes, oxidation and reduction reactions take place in the electrodes when two dissimilar electrodes are combined with a dilute electrolyte. One electrode, known as the cathode, acquires a negative charge as a result of the oxidation reaction. Due to the reduction reaction, an additional electrode is given a positive charge and is referred to as the anode. A battery's negative terminal is created by the cathode, whereas the positive terminal is created by the anode. Energy can be stored in a chemical form in rechargeable storage systems, which are practical energy storage devices. There are numerous battery types available today, each with unique concepts that fit a particular need. These developments frequently include a variety of features, such as different sizes and chemical components built into them. Battery technology has advanced significantly in recent years, particularly with regard to Li-ion batteries (LIBs) [141, 142]. LIBs are now widely utilized in small electronic devices, renewable energy sources, and microgrid systems. Depending on the electrolytic system, LIBs have a high energy storage efficiency and attractive characteristics such as low maintenance requirements, extended cycle lives, and high design flexibility. A schematic representation of the interior structure of LIB is shown in Fig. 14. The most adaptable electrolytes are aqueous ones, but they have many disadvantages, such as low heat stability and a propensity to quickly degrade into hazardous chemicals [143]. The demand for batteries in electric vehicles and smart grid applications is a key factor in the commercialization of batteries. Apart from price variances, each battery type differs from the others in terms of operating performance and attributes. As a result, one type of battery can be especially suitable for a certain storage function. Therefore, the energy storage business will greatly benefit from a range of battery technologies.

### MXenes for supercapacitors

MXene inks can be converted into 2D films of MXene (Ti_3_C_2_T_*x*_) for improved electron transport when making electrodes for supercapacitors. The breakdown of MXene components and oxidative instability when in contact with water are the key issues preventing its practical implementation. The MXene composite can have tartaric acid added to it to prevent oxidation and firmly keep the assembly components together [144]. By using the laser crystallization process to create sandwiched electrodes (for example, MXene/Fe_3_O_4_/MXene), the device's areal capacitance and cycle stability will be improved [145]. The structural and electrochemical features of 2D MXene can be changed by annealing treatment [146]. Due to architectural and chemical changes on the surface of the MXene, direct annealing processes aid in improving the strength of the structures, electrochemical performances, and cycle stability. This also promotes ion movement and enhances electrolyte accessibility for high-performance supercapacitors [147].

There is currently significant research into finding new and high-capacitance MXene-based SC electrode materials. 2D MXenes such as Ti_3_C_2_, Mo_2_C, Ti_2_C, and Mo_1.33_C have demonstrated enhanced electrochemical features as SC electrodes. By selectively etching Al from vanadium aluminum carbide (V_4_AlC_3_), a multilayered 2D vanadium carbide (V_4_C_3_) MXene was synthesized for SC electrode material [148]. The synthesized material exhibited strong rate performance, high capacitance (209 F/g at 2 mV/s), and stable extended cyclic performance. The large pore volumes and specific surface areas, strong hydrophilicity, and the numerous valence states of the vanadium contributed to the excellent capacitance of the V_4_C_3_ MXene. The good electronic conductivity of the synthesized 2D electrodes is primarily responsible for their superior cycle stability and enhanced rate performance. Another work also presented a thorough examination of the characteristics and the electrochemical performance of a synthesized V_4_C_3_ MXene as a potential supercapacitor electrode [149]. By utilizing Hydrofluoric acid etching to remove the intermediary "A-Aluminum" layer from the MAX phase, the 2D MXene was created. The synthesized samples demonstrated a layered structure, good electrochemical performance, and device stability for SC applications. This performance can be attributed to the inherent features of the vanadium atom.

**Fig. 15:**
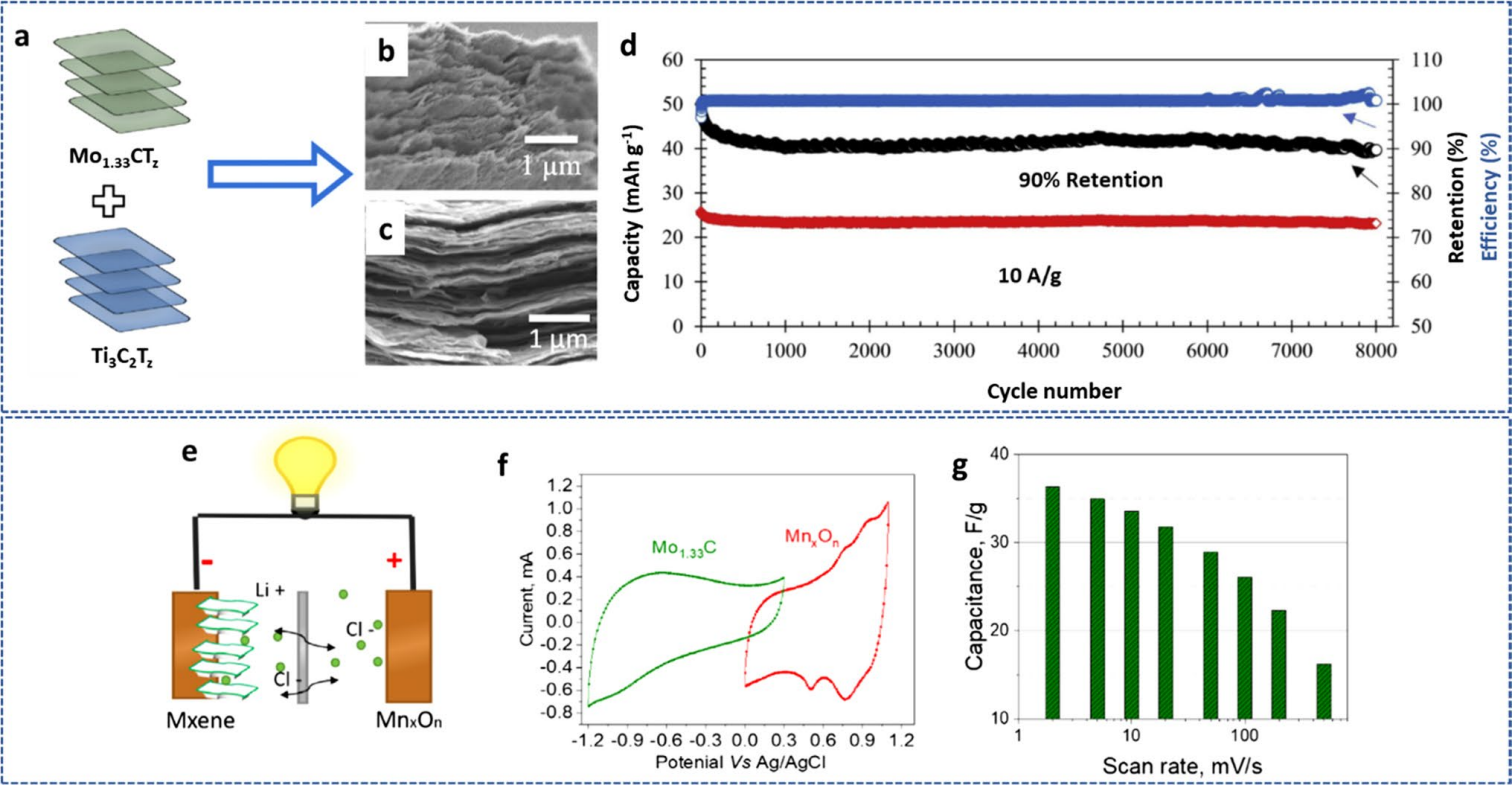
**a** Schematic fabrication of the electrodes. **b** and **c** SEM images of the thin and thick, respectively. **d** Long-term cycle performance at 10 A/g for 8.0-micron-thick electrodes. Adapted from reference [150], Copyright (2021) by the authors. Published by Elsevier Ltd. This article is an open-access article distributed under the terms and conditions of the Creative Commons Attribution (CC BY) license. **e** Schematic illustration of Mo_1.33_C/Mn_*x*_O_*n*_ design **f** Capacitance of MXene//Mn_*x*_O_*n*_ device using different scan rates. **g** Ragone plot of Mo_1.33_C/Mn_*x*_O_*n*_ compared with some MXene-based devices. Adapted with permission [151], Copyright (2021) by the authors. Published by Elsevier B.V. This article is an open-access article distributed under the terms and conditions of the Creative Commons Attribution (CC BY) license

The investigation of sustainable energy storage systems, such as Zn-ion hybrid SCs (HSCs), is motivated by the rising demand for quickly rechargeable batteries and SCs as well as the scarcity of their active components (such as Li and Co). Etman et al*.* [150] reported how Zn-ion HSCs were used as freestanding Mo_1.33_CT_z_–Ti_3_C_2_T_z_ coupled MXene films (Fig. 15a). One-step vacuum filtration was employed to create the mixed MXene films from pure MXene suspensions. The mixed MXene produced capacities of around 159 mAh/g at 0.5 mV/s scan rate. Additionally, after 8000 cycles, the electrodes showed a promising 90% capacity retention as shown in Fig. 15d. The mixed MXene also recorded energy densities of around 103 Wh/kg at power densities of 0.143 kW/kg. The above performance can be attributed to in-plane ionic transport which resulted in an increased number of the available electrochemically active sites as displayed by the SEM images in Fig. 15b,c. 2D Mo_1.33_C MXene is mostly researched in the H_2_SO_4_ electrolyte medium and exhibits tremendous promise for energy storage applications. The electrode potential is nevertheless restricted by H_2_SO_4_ to about 0.9 V and 1.3 V for symmetric and asymmetric devices, respectively. Ghazaly and co-researchers [151] investigated Mo_1.33_C MXene's electrochemical performance in a LiCl electrolyte, which is less hazardous compared to H_2_SO_4_. According to the research, the operational potential window for the device (vs. Ag/AgCl) was a wide − 1.2 to + 0.3 V, with a volumetric capacitance of 815 F/cm^3^ at 2 mV/s. This was subsequently utilized to create MXene-based asymmetric SCs (Mo_1.33_C/Mn_*x*_O_*n*_) (Fig. 15e), and the performance of the electrochemical system was assessed in a 5 M LiCl electrolyte. Figure 15f shows CV curves of the positive and negative electrodes at 10 mV/s. A volumetric energy density of 58 mWh/cm^3^ and a maximum power density of 31 W/cm^3^ were attained, thanks to the broad voltage widow of the Mo_1.33_C/Mn_*x*_O_*n*_ devices. Figure 15g shows the gravimetric capacitance of MXene//Mn_*x*_O_*n*_ device at different scan rates. 2D V_2_CT_*x*_ MXene was also created by selectively etching layers of Al from the V_2_AlC MAX phase using a NaF + HCl etching solution at 90 °C for 72 h. The performance of the resulting material as an SC electrode was then evaluated using an electrolyte that mimicked seawater. An excellent capacitance (181.1 F/g) was displayed by synthesized electrodes with 89.1% of the initial capacitance retained even after 5000 cycles [152].

**Fig. 16:**
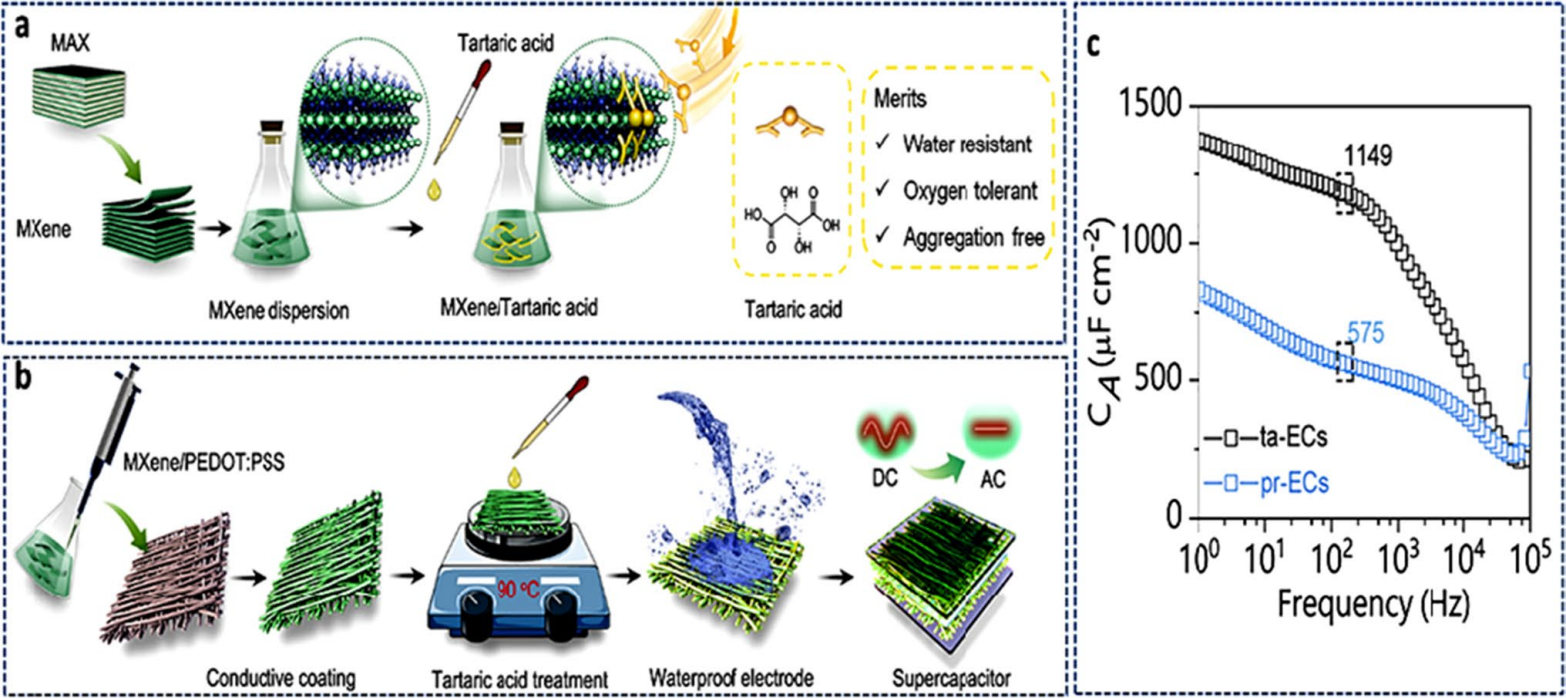
**a** Chemical stabilization of Ti_3_C_2_T_*x*_ using tartaric acid. **b** Structural stabilization of Ti_3_C_2_T_*x*_/PEDOT:PSS composite for an ultrafast SC. **c** Areal capacitance (*C*_A_) variation with frequency for ta-EC and pr-EC. Adapted from reference [144], Copyright (2021) by the authors. Published by Elsevier. This article is an open-access article distributed under the terms and conditions of the Creative Commons Attribution (CC BY) license

Ti_3_C_2_T_*x*_, a flourishing 2D MXene material, has demonstrated growing promise in a variety of applications, including printed electronics and energy storage. However, its practical application has been constrained by its oxidative instability and the ease with which its assemblies disintegrate when in contact with water. The advantages of tartaric acid, a substance derived from a natural source, as an additive component in the MXene composite have been described (Fig. 16a) [144]. In addition to keeping individual components of the composite Ti_3_C_2_T_*x*_/PEDOT:PSS (Ti_3_C_2_T_*x*_ blended with polymer poly-3,4-ethylene dioxythiophene (PEDOT): polystyrene sulfonate (PSS)) tightly together and inhibiting Ti_3_C_2_T_*x*_ oxidation in water, it also increased the composite's electron conductivity compared to the additive-free setup. The composite material was used in real-life applications as a super-fast SC which delivered superb electrochemical performance as shown in Fig. 16b,c. Tantalum carbide MXene film was synthesized by hydrofluoric acid etching of the intermediate Al from the Ta_4_AlC_3_ MAX phase [153]. After etching, the absence of Al rendered the intermediate "A" element invisible, but FESEM analysis revealed that the synthetic tantalum carbide had the morphology of layered solid structures. In addition, the synthesized MXene was investigated for its electrochemical performance in energy storage applications. The produced MXenes were useful for allowing electrolytic ions to enter the electrode, boosting the electrochemical activity. The electrochemical testing revealed a maximum specific capacitance (481 F/g at 5 mV/s) with a high cyclic retention rate (about 89%) even after 2000 cycles in 0.1 M H_2_SO_4_ electrolyte. These results were obtained due to the fast electrolytic ion transport. Lignosulfonate (LS) P-π conjugate architectures give α and β carbon a good local positive potential and chemical reactivity, which can be used to change the surface of MXene and prevent the restacking issue. A modified LS MXene (Ti_3_C_2_T_*x*_-rGO) 3D porous aerogel was synthesized for the first time [154]. Even with a high mass loading (5.1 mg/cm^2^), the synthesized aerogel showed superior electrochemical performance when compared to pure MXene. As an SC electrode, it displayed high-rate performance and a specific capacitance of 386 F/g at 2 mV/s. Additionally, it is claimed that the 3D functionalized porous LS rGO aerogel matches with the synthesized Ti_3_C_2_T_*x*_-rGO aerogel to create an all-pseudocapacitive asymmetric SC under positive potential. As a result, the asymmetric SC supplied 142 µWh/cm^2^ of energy (at 4900 W/cm^2^ power), maintaining 96.3% of the initial capacitance after 10,000 charge/discharge cycles. These results can be linked to the interconnected porous structure of the prepared material which aided ionic transport.

**Fig. 17:**
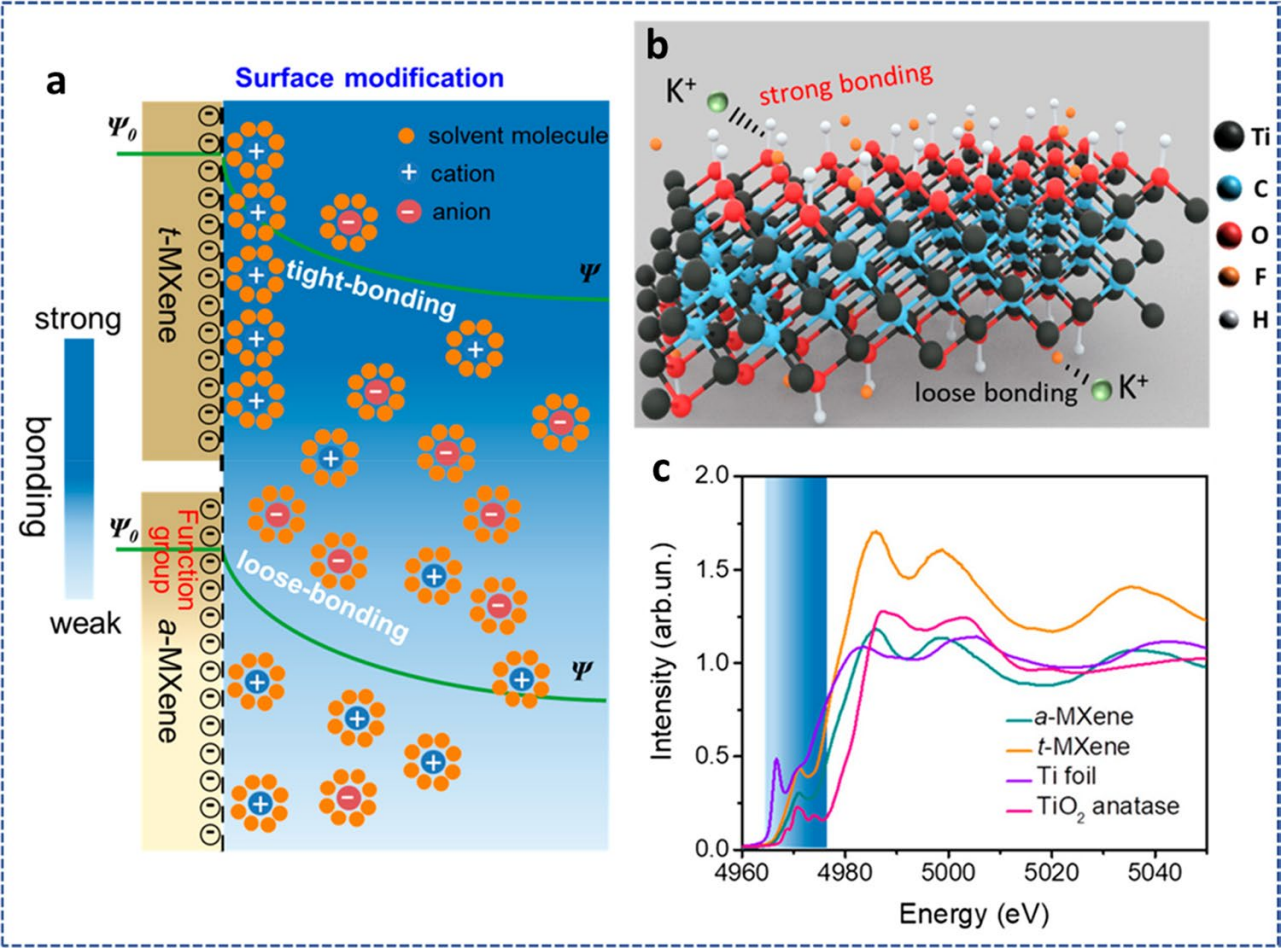
**a** Schematic illustration of the various tight-bonding models. **b** Schematic illustration of the suppressing self-discharge via variation of surface functional groups. **c** X-ray absorption of the various materials. Adapted with permission [155], Copyright (2020), American Chemical Society

MXenes' improved electrochemical activity is facilitated by their surface functionalization and rich chemistry, but this also significantly exacerbates its self-discharge behavior in SCs. However, there are still problems with this self-discharge habit and its associated mechanism. The self-discharge nature of Ti_3_C_2_T_*x*_ MXene-based SCs was successfully uncovered, and a chemically interfacial-tailored regulating technique was proposed to effectively alleviate it (see Fig. 17a). The interaction of ionic counterparts was strongly impacted by the significant alteration of the oxidation state and coordination features on the MXene, which was clearly shown by X-ray absorption structures. Theoretically, greater adsorption energy existing between the electrode/electrolyte interface was shown to be the cause of this much better self-discharge (Fig. 17b, c) [155].

**Fig. 18:**
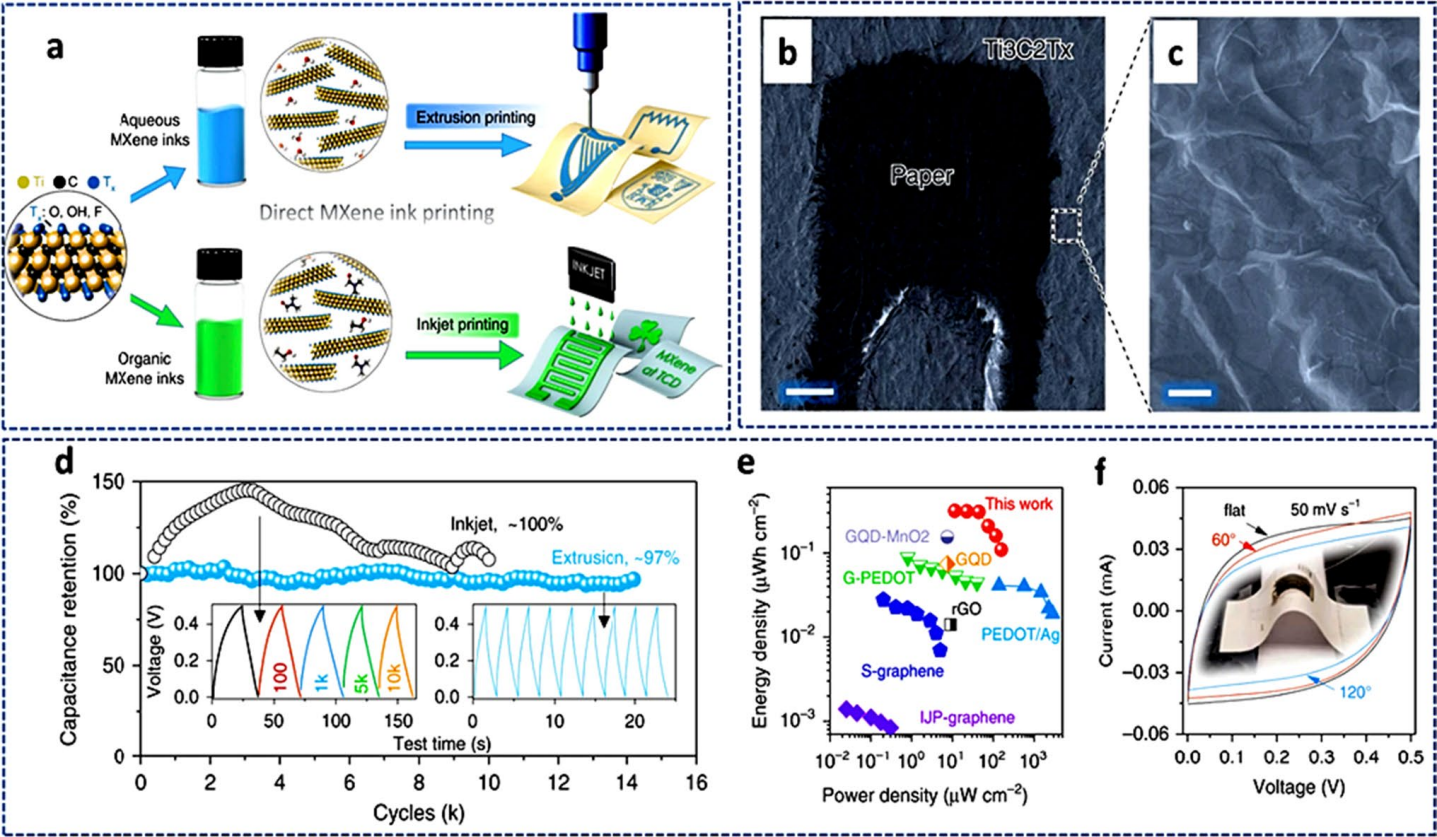
**a** Schematic illustration for the direct MXene ink printing. **b** and **c** Low- and high-magnification SEM images of the printed MXene MSC. **d** Long-term cycle test of the inkjet/extrusion-printed MSCs. **e** Ragone plot comparison of different MSC device systems. **f** CVs of extrusion-printed MSC supported on a paper substrate (inset) under different bending degrees. Adapted from reference [156]. Copyright (2019) by the authors. Published by Springer Nature. This article is an open-access article distributed under the terms and conditions of the Creative Commons Attribution (CC BY) license

Functional inks must be printed directly for use in numerous industries, such as healthcare, smart electronics, and electrochemical energy storage. The compositions of printable ink that are now on the market are far from perfect. Surfactants and other additives are frequently used, or the concentrations of the ink are usually low, which complicates manufacturing and lowers printing resolution. Typically, an additive is necessary to hold the particles together and allow for high-quality printing in most other nano-inks, but in the case of MXenes, no addition is required; simply use MXene in water or MXene in an organic solution to generate the ink [156]. For instance, a group of researchers presented two different kinds of 2D Ti_3_C_2_T_*x*_ MXene inks, organic and aqueous, for inkjet and extrusion printing, respectively, without any additives as shown in Fig. 18a [156]. The fabricated device was employed as a micro-SC with interconnected, stacked nanosheets depicted in Fig. 18b,c. In comparison with current extrusion/inkjet-printed materials, the energy density and volumetric capacitance of the micro-SCs were of higher magnitude (Fig. 18d,e). The adaptable direct-ink-printing method demonstrates the potential of MXene inks without additives for the mass production of simple SC components. Figure 18f shows CVs of extrusion-printed MSC supported on a paper substrate (inset) under different bending degrees. The electrochemical performance of some MXene-based materials in supercapacitors is shown in Table 2.

**Table 2:** Performances of MXene-based electrodes in SCs

MXene-based materials	Method	wt. (%)	Capacitance (F/cm^3^@mV/s)	Rate cap. (%)	Range (mV/s)	Cycle no	Rc (%@mV/s)	Refs.
Ti_3_C_2_T_*x*_	HF	2 − 3 (mg/cm^2^)	340@20	62.2	2 − 100	10^4^	100/5	[157]
d-Ti_3_C_2_T_*x*_	HF	1.2 (mg/cm^2^)	520@2	42.3	2 − 100	10^4^	100/5	[158]
Ti_3_C_2_T_*x*_ hydrogel	HCl/LiF	5.3 (mg/cm^2^)	1500@2	56.8	2 − 10^4^	10^4^	90/5	[159]
Ti_3_C_2_/PVA-KOH	VAF	90	528@2	58.5	2 − 10^2^	10^4^	83.7@5 A/g	[124]
Ti_3_C_2_/PDDA	VAF	90	296@2	74.3	2 − 10^2^	10^4^	88.7@5 A/g	[124]
Ti_3_C_2_/PPy	VAF	66.7	1000@5	–	2 − 10^5^	2.5 × 10^4^	92@100	[118]
Ti_3_C_2_/rGO/PDDA	VAF	95	1040@2	61	2 − 10^3^	2.5 × 10^4^	∼100@100	[86]
MWCNT/Ti_3_C_2_/PCL	SC	24	182 F/g@10	15	10 − 10^5^	∼2100	80	[160]
Ti_3_C_2_/PEDOT	ED	–	2.4 mF/cm^2^@10	85.3	10 − 10^6^	3 × 10^4^	∼100	[161]

*PVA* poly(vinyl alcohol), *PDDA* polydiallyldimethylammonium chloride, *PPy* polypyrrole, *PCL* polycaprolactone, *MWCNT* multiwalled carbon nanotube, *PEDOT* poly(3,4-ethylenedioxythio-phene), *PDA* polymeric dopamine, *VAF* vacuum-assisted filtration, *ED* electrochemical deposition, *SC* spray coating

### MXenes for batteries

Due to its exceptional physical and electrochemical capabilities, numerous surface chemistries, and distinctive 2D structures, MXenes have emerged as a research material of choice for metal–ion, metal–air, and metal–S batteries. The following subsections discuss the synthesis techniques, structure, and property relation of MXene-based battery production.

#### MXene for metal–ion batteries (MIBs)

**Fig. 19:**
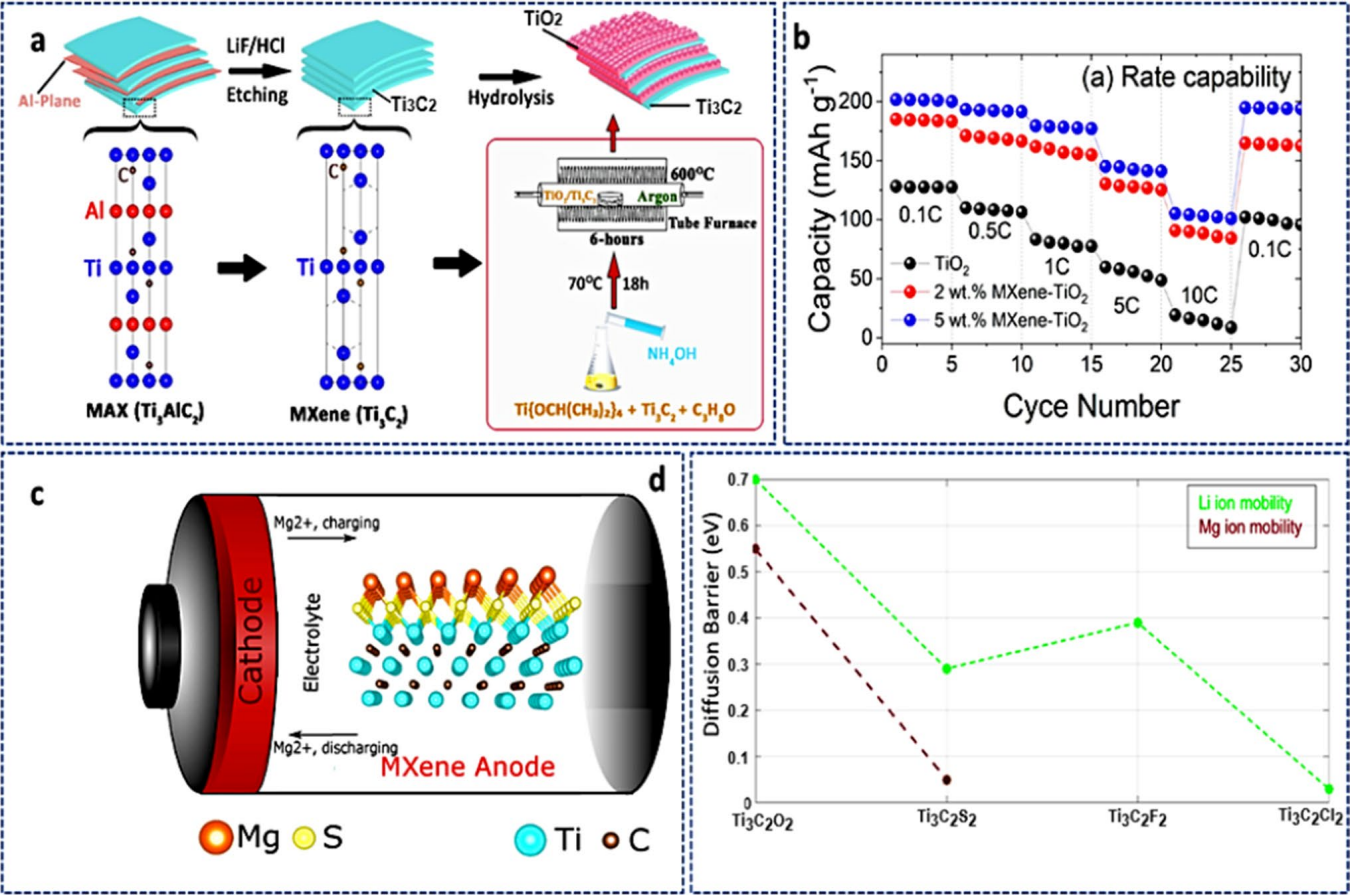
**a** Schematics for the synthesis of MXene–TiO_2_ materials utilizing Ti_3_AlC_2_ and titanium isopropoxide as a precursor for TiO_2_. **b** Rate capability of TiO_2_ and MXene–TiO_2_ at room temperature. Adapted from reference [163], Copyright (2022) by the authors. Published by Elsevier B.V. This article is an open-access article distributed under the terms and conditions of the Creative Commons Attribution (CC BY) license. **c** Operations of a MIB battery using a MXene anode. **d** The migration energy barriers for MIB and LIB using MXene-based anodes. Adapted from reference [164], Copyright (2022) by the authors. Published by Elsevier B.V. This article is an open-access article distributed under the terms and conditions of the Creative Commons Attribution (CC BY) license

Since some firms began selling metal–ion batteries, they have attracted a lot of attention as the most advanced component of electrochemical energy storage systems, particularly batteries. Anode, cathode, separator, and electrolyte are the four main components of a standard MIB. The cathode undergoes a reduction process during charging, which produces free ions. These metal ions then travel through the separator and are inserted into the anode via the electrolyte. Metal ions are released from the anode during discharge, and they travel through the separator to the cathode for possible oxidation. Several MIBs have been studied in the last decade, including Li, K, Mg, Zn, etc. Utilizing the fact that sulfur (S) terminations lower the ions' diffusion barriers, Papadopoulou et al*.* [162] recently explored Li, Zn, Mg, and K ion intercalation for the first time on the surface of a monolayer of Zr_2_CS_2_ MXene. The researchers discovered that the structures of Zr_2_CS_2_–Li, Zr_2_CS_2_–Mg, and Zr_2_CS_2_–K were all the same, except for Zr_2_CS_2_–Zn, which differed in the position of ion and Zn detachment from the surface of the MXene during migration. Regarding the usage of Zr_2_CS_2_ as an anodic material in MIBs, they investigated as criteria the energy for adsorption and open-circuit potential for the various ions studied. It was demonstrated that K ion revealed lower open-circuit voltage and increased mobility. These findings indicate that when S-terminated, Zr-based materials were used as anode electrodes, KIB had higher energy density and faster charge–discharge rates than the others. Therefore, KIB appears to be the best substitution for LIB, especially when K's low cost and wealth of resources are taken into account. For high-power LIBs, TiO_2_ might make good anodic material. Its practical applicability is nevertheless constrained by its weaker electronic conductivity. In situ hydrolysis was used to create a new TiO_2_ anode decorated with Ti_3_C-MXene for LIBs as shown in Fig. 19a [163]. The electrical and structural properties were enhanced by utilizing MXenes as a base material because of their well-known exceptional structural stability and good electronic conductivities. Additionally, the electrochemical impedance spectroscopy study showed that MXene–TiO_2_ composite materials had quicker kinetics than the pristine TiO_2_ anode. The anodic material recorded a capacity of about 200 mAh/g at 0.1C (see Fig. 19b), making it possible to be used in high-performance LIBs due to its exceptional electrochemical performance. The recorded results could be attributed to the large surface area created which yielded more active sites for Li-ion intercalation.

Recently, the diffusion and adsorption of an Mg ion on the surface of Ti_3_C_2_S_2_ MXene were examined using Density Functional Theory to determine the structure of the compound [164]. In the Ti_3_C_2_-based materials, very high adsorption and a low energy barrier for Mg diffusion were recorded as compared to other metal ions (Fig. 19c,d). Therefore, the Ti_3_C_2_S_2_ MXene could be employed ultimately as an ideal choice for an anodic electrode in Mg-ion batteries, providing a safer, more affordable alternative to LIBs. Sun et al*.* [165] made a forecast on a 2D Ti_3_C_3_ with a sandwiched-tetragonal structure, based on first-principles calculations and evolutionary search. This material was discovered to be more stable than the well-known MXene Ti_3_C_2_. A high Young's modulus, a good fracture strain, and high fracture stress are among the mechanical characteristics that outperform other materials, including graphene. Metal–ion batteries have a lot of potential thanks to excellent metallic characteristics. Li, Na, and K have the lowest diffusion barriers and high diffusibilities, which permit quick charge and discharge. These properties make it possible in the application of storage devices. The electrochemical performance of some MXene-based materials in metal–air batteries is shown in Table 3.

**Table 3:** Electrochemical performance of some MXene-based metal–air materials in batteries

Electrode	Capacity/mAhg^−1^	Rate (mA/g)	Remarks	Refs.
Nb_2_O_5_@Nb_4_C_3_T_*x*_	208	50	94% retained after 400 cycles	[166]
TiO_2_/Ti_3_C_2_T_*x*_	267	0.2	Dropped and regained after 2000 cycles	[167]
TiO_2_/Ti_3_C_2_T_*x*_	124	50	Retained after 400 cycles	[168]
Ti_3_C_2_T_*x*_/Co_3_O_4_	50	20	Retained after 100 cycles	[169]
Fe_3_O_4_@Ti_3_C_2_T_*x*_	278	5	Measured after 800 cycles	[170]
PVP-Sn(IV)@Ti_3_C_2_T_*x*_	544	0.5	Measured after 200 cycles	[80]
SnO_2_/Ti_3_C_2_T_*x*_	400	0.1	360 mAhg^−1^ after 200 cycles	[171]
MoS_2_/Mo_2_TiC_2_T_*x*_	90	5	92% retained after 100 cycles	[172]
MoS_2_/Ti_3_C_2_@C	580	20	95% retained after 3000	[173]
Ti_3_C_2_T_*x*_/NiCo_2_O_4_	1330	0.1	Retained after 100 cycles	[169]

#### MXene for metal–sulfur batteries (MSBs)

The production costs and poor specific capacities of the usual cathode materials, such as layered-metal oxides (e.g., LiCoO_2_) and spinel structures (e.g., LiNi_2_O_4_), limit their utilization. Additionally, the anodes in MIBs made of carbonaceous materials have a low specific capacity. As a result, MIBs have low energy densities, which further prevents their use in smart grids, electronics, and electric vehicles. Therefore, the demand for new battery technologies with better energy densities is important. Due to the low price and extraordinarily high energy densities, metal–sulfur batteries (MSBs) stand out among the alternatives as prospective candidates for the next generation of advanced energy storage technologies. The ring-structure octasulfur (S_8_) cathode in MSBs is an essential component, especially in lithium–sulfur battery (LSB) systems [174].

Pai et al*.* [175] showed for the first time that confined sulfur (S_8_) molecules can be contained in carbonate electrolytes in alkali metal (such as Li, Na, K)–sulfur systems by using high-conductive Ti_3_C_2_T_x_ MXene sheets as a source. This quasi-solid-state process had many benefits over traditional liquid-phase electrochemical reactions in Li–S, which could open up new possibilities for the design and manufacturing of metal–S battery materials in the future. In this work, alkali metal sulfur batteries at room temperature are a possible replacement for LiBs due to their large capacities and low intrinsic cost. The multilayered-MXene structure displayed a unique interlayer spacing that inhibits unfavorable polysulfide–carbonate interactions and offers variable spacing for the S_8_ confinement. A potential energy storage technology, such as LSBs, has been considered recently due to its high energy density and theoretical capacity. However, a solution still needs to be found for the irritating shuttle effects and weak electronic/ionic conductivity. A sandwich-shaped Ti_3_C_2_T_*x*_ MXene interlayer with good flexibility and conductivity has been reported with TiS_2_/TiO_2_ surface heterostructure [176]. Lithium polysulfides (LiPSs) were combined using TiO_2_ nanoparticles as an adsorbent and TiS_2_ nanoparticles as catalysts to further speed up the conversion of LiPSs long-chain to Li_2_S_2_/Li_2_S short-chain. The interlayer also shielded the anodic Li metal by preventing the buildup of polysulfide on its surface. The fabricated composite device performed superbly with consistent long-term cycling when used as an LSB.

**Fig. 20:**
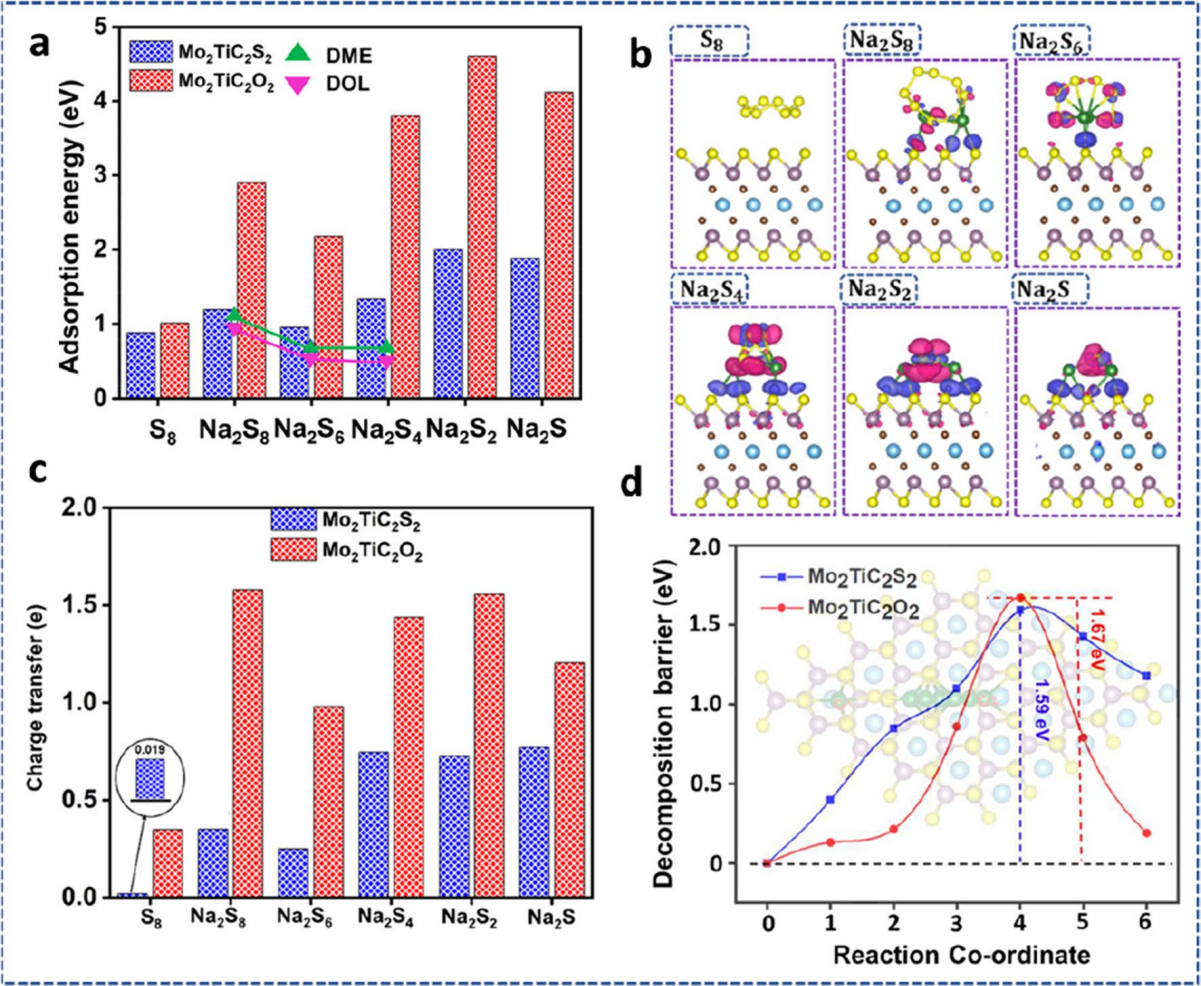
**a** Calculated adsorption energies of the adsorbed S_8_ and Na_2_Sn on Mo_2_TiC_2_O_2_ and Mo_2_TiC_2_S_2_. **b** Side view differential charge density calculation of S_8_ and Na_2_Sn on Mo_2_TiC_2_S_2_ substrate. **c** Charge transfer of adsorbed S_8_ and Na_2_Sn on Mo_2_TiC_2_T_2_. **d** Computed decomposition barriers of Na_2_S on the substrates of Mo_2_TiC_2_O_2_ and Mo_2_TiC_2_S_2_. Adapted with permission [178], Copyright (2022), American Chemical Society

To achieve a high volumetric capacity of LSBs without compromising gravimetric performance, a cathode with dense and high S-loading with limited porosity is greatly needed. However, the sluggish S-redox kinetics and extensive shuttling greatly limit its development. A heterostructure consisting of a bi-conductive 1T-VS_2_-MXene electrocatalyst that functions as a highly efficient sulfur host has been reported in recent times [177]. It combines the advantages of ultrafast anchoring and catalysis (function of the 1T-VS_2_) with good surface diffusion and nucleation (function of the MXene) to speed up the bidirectional S-redox kinetics. The fabricated monolith cathode obtained a high volumetric capacity of a typical electrolyte at 0.1C and a significant areal capacity in an exceptionally lean electrolyte. From the standpoint of the atomic and electronic structure, theoretical and experimental results also demonstrated that the source of higher performance was due to the synergistic effects of the heterostructured catalyst. In addition, Nahian et al*.* [178] used DFT calculations to examine how Na_2_Sn interacts with Mo_2_TiC_2_O_2_ and Mo_2_TiC_2_S_2_ and assess how well these materials function as the anchoring materials (AMs) for sodium–sulfur batteries (NaSBs). Calculated S_8_/Na_2_Sn species adsorption energies on both MXenes revealed that Mo_2_TiC_2_S_2_ had a moderate adsorption strength compared to Mo_2_TiC_2_O_2_ as shown in Fig. 20a. Notably, neither Na_2_Sn nor Mo_2_TiC_2_S_2_ species experienced considerable structural deformation, which is necessary to reduce capacity fading and increase battery cyclability, despite appropriate bond formation between the substrates and Na_2_Sn. Using Bader charge and charge density differential studies, the researchers were able to better correlate the Na_2_Sn anchoring process on the AMs (see Fig. 20b). A significant degree of charge transport occurred from Na_2_Sn to Mo_2_TiC_2_T_2_ (Fig. 20c), subsequently, the development of covalent bonds within the structure caused an increase in adsorption strength in the insoluble lower-ordered polysulfides. Mo_2_TiC_2_S_2_ was predicted to efficiently limit Na_2_Sn shuttling and dissolution without causing Na_2_Sn to decompose (Fig. 20d) because it displayed a moderate adsorption strength as compared to Mo_2_TiC_2_O_2_.

### MXenes for flexible electrochemical energy storage devices

#### MXene for flexible supercapacitors

**Fig. 21:**
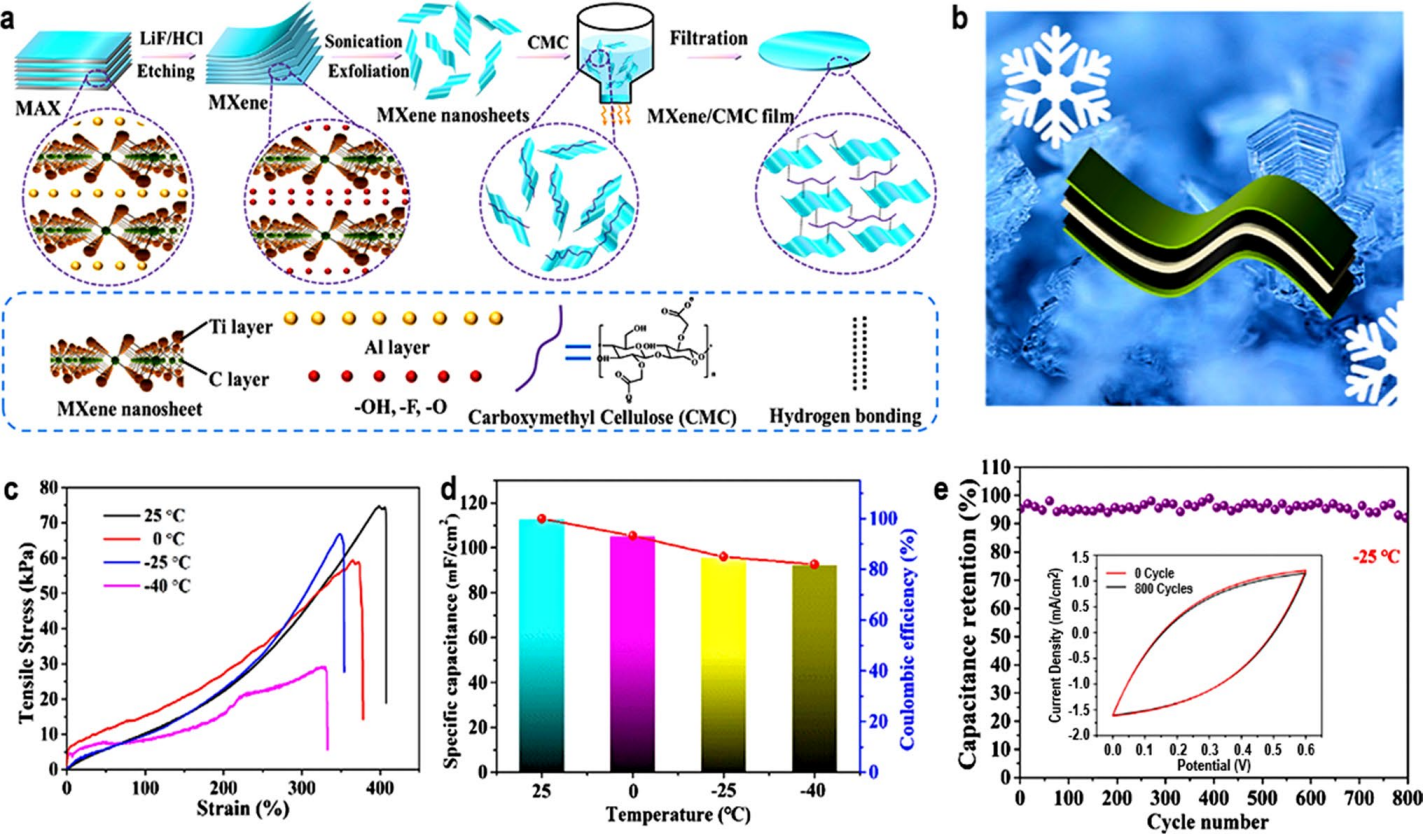
**a** Schematic preparation of MXene/CMC film. **b** Schematic of the flexible SC under low temperature. **c** Tensile tests of the PVA/LiCl hydrogel (original and healed) at different self-healing times. **d** Capacitance retention of the SC under different temperatures. **e** Cycle performance of the SC 25 °C. Adapted with permission [179], Copyright (2022) by the authors. Published by Elsevier. This article is an open-access article distributed under the terms and conditions of the Creative Commons Attribution (CC BY) license

Flexible devices have demonstrated great applications in a variety of fields, including flexible displays, electronic skin, human–machine interfaces, smart monitors, etc. This has raised the requirements for the innovation of effective power sources to power these wearable electronics to new heights. Due to the quick charge–discharge capacity and strong cyclic stability, supercapacitors are one of the most promising power units among other energy storage devices. Supercapacitors must have mechanical properties compatible with human skin to be used as wearable electronics power sources. This means that the supercapacitors must not only be able to supply continuous power under varied deformation brought on by human motions, but also be comfortable for the wearer. The development of solid-state supercapacitors with high mechanical flexibility has received a lot of attention, but it is still far from adequate for wearable applications. Because of the electrode rigidity, electrolyte freezing, and the issues with interfacial contact, creating a flexible all-solid supercapacitor that can function under varied deformations, even under harsh conditions, remains difficult. Recently, Yin et al*.* [179] provided an assembly of a flexible SC with superior mechanical deformation, ultra-low temperature forbearance, and a hydrogel of polyvinyl alcohol/LiCl (PVA/LiCl) electrolyte. The flexible SC was designed from the cellulose film of the MXene/carboxymethyl (MXene/CMC) electrode (Fig. 21a). The SC combined highly conductive and physically flexible electrodes with hydrogel electrolyte that has self-adhesion, exceptional mechanical characteristics (see Fig. 21b, c), and antifreezing capabilities. The built supercapacitor device displayed a high specific capacitance of 113.13 mF/cm^2^ with the device retaining almost 95% during severe mechanical deformations as shown in Fig. 21d, e.

**Fig. 22:**
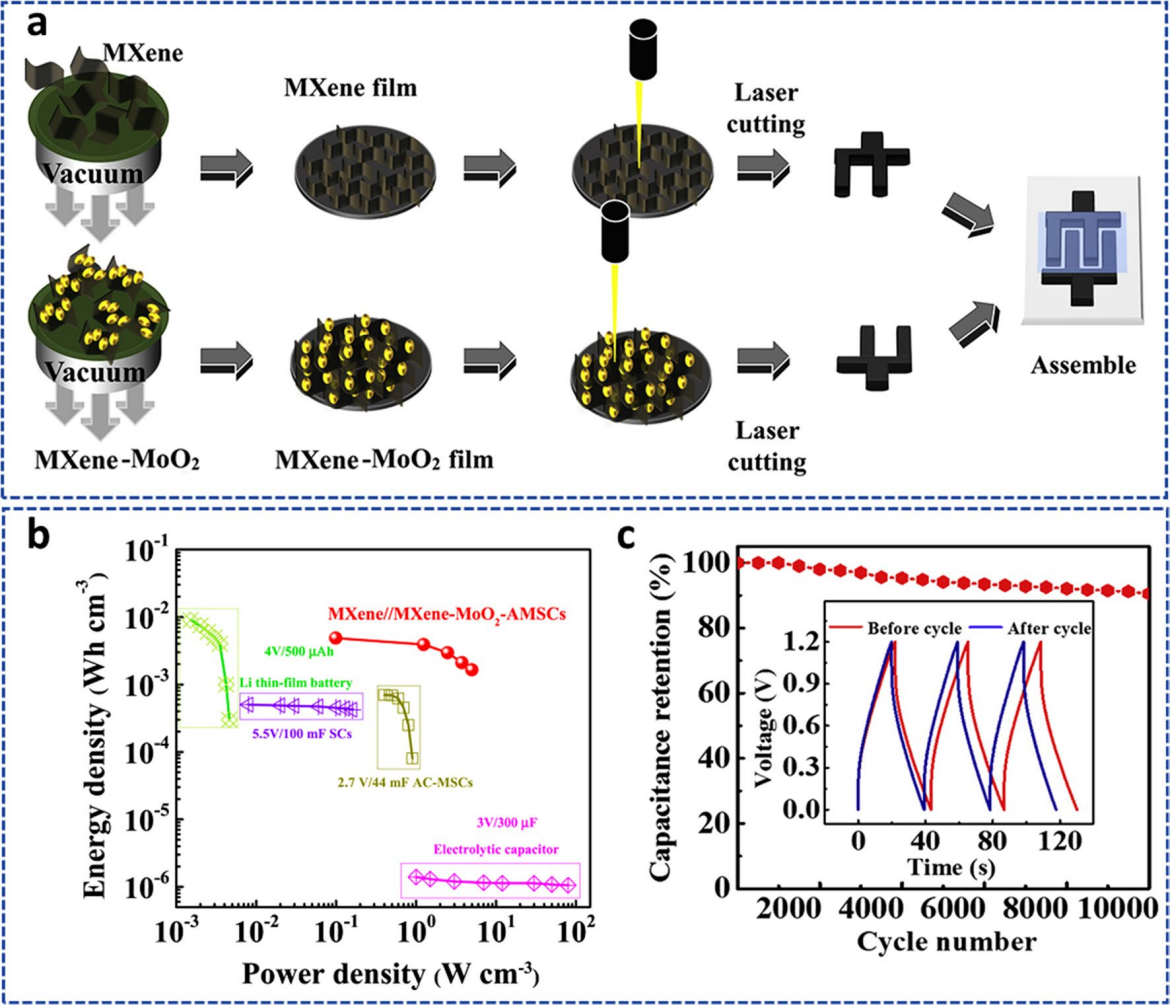
**a** Schematic of the fabrication process of MXene//MXene-MoO_2_-AMSCs. **b** Ragone plot of in-plane MXene//MXene-MoO_2_-AMSCs compared with commercially available energy storage devices. **c** Cycling stability of MXene//MXene-MoO_2_-AMSCs. Adapted with permission [180], Copyright (2020) by The Chinese Ceramic Society. Production and hosting by Elsevier B.V. This article is an open-access article distributed under the terms and conditions of the Creative Commons Attribution (CC BY) license

Micro-supercapacitors (MSCs) using 2D MXenes offer significant potential. However, due to the oxidation at high anodic potentials, the maximum output voltage of symmetric MXene MSCs is constrained. Zhang et al*.* [180] presented the development of asymmetric MSCs (AMSCs) using MXene-MoO_2_ and Ti_3_C_2_T_*x*_ electrodes. To prevent layer stacking and nanoparticles of MoO_2_ aggregation, 0D MoO_2_ nanoparticles were homogeneously dispersed into MXene layers to create the 2D-0D MXene-MoO_2_ microelectrode (Fig. 22a).

The AMSCs displayed good electrochemical performances, including high energy density (9.7 mWh/cm^3^) at a power density of 0.198 W/cm^3^) (Fig. 22b), and volumetric capacitance. With about 88% of the initial capacitance retained after 10,000 cycles (Fig. 22c), the AMSCs also demonstrated great mechanical flexibility under various bending tests and excellent cycling stability. A very effective supercapacitor was built using a flexible hybrid film of N-doped 3D reduced graphene oxide (N-RGO)/CNT-MnO_2_. The resulting hybrid film had impressive capacitance retentions of 95% using aqueous electrolytes after undergoing 5000 cycles. It also had substantial specific capacitances of 418 F/g. The synthesized flexible hybrid film was used to create a basic supercapacitor. Under a scan rate of 50 mV/s, the built flexible supercapacitor device demonstrated a high energy density of 45.72 Wh/kg and simultaneously sustained a huge power density of 12,526 W/kg. The recorded performance can be attributed to the interconnected porous structure created by the 3D architecture which aided in electrolytic ion transport [181]. The electrochemical performance of some MXene-based flexible SCs is shown in Table 4.

**Table 4:** Electrochemical performance of some MXene-based flexible SCs

Electrode	Electrolyte	Capacitance	Stability	Refs.
Ti_3_C_2_T_*x*_ films	1 M H_2_SO_4_	245 F/g at 2 mV/s	100% after 10,000 cycles	[182]
Ti_3_C_2_T_*x*_-Li film	1 M H_2_SO_4_	892 F/cm^3^ at 2 mV/s	100% after 10,000 cycles	[183]
MXene/rHGO	3 M H_2_SO_4_	1445 F/cm^3^ at 2 mV/s	93% after 10,000 cycles	[184]
MXene/rGO-5 wt.%	1 M KCl	1040 F/cm^3^ at 2 mV/s	100% after 20,000 cycles	[86]
MXene/graphene	3 M H_2_SO_4_	127 F/g at 2 mV/s	95.7% after 10,000 cycles	[185]
layered Ti_3_C_2_/PPy	PVA/H_2_SO_4_	35.6 mF/cm^2^ at 0.3 mA/cm^2^	100% after 10,000 cycles	[186]
Ti_3_C_2_T_*x*_/PANI	1 M H_2_SO_4_	272.5 F/g at 1 A/g	71.4% after 4000 cycles	[187]
Ti_3_C_2_T_*x*_/PEDOT:PSS	1 M H_2_SO_4_	1065 F/cm^3^ at 2 mV/s	80% after 10,000 cycles	[188]
Mo_1.33_C MXene/PEDOT:PSS	1 M H_2_SO_4_	1310 F/cm^3^ at 2 mV/s	90% after 10,000 cycles	[189]
Ti_3_C_2_T_*x*_/MnO_2_	1 M Na_2_SO_4_	130.5 F/g at 0.2 A/g	100% after 1000 cycles	[190]

#### MXene for flexible batteries

**Fig. 23:**
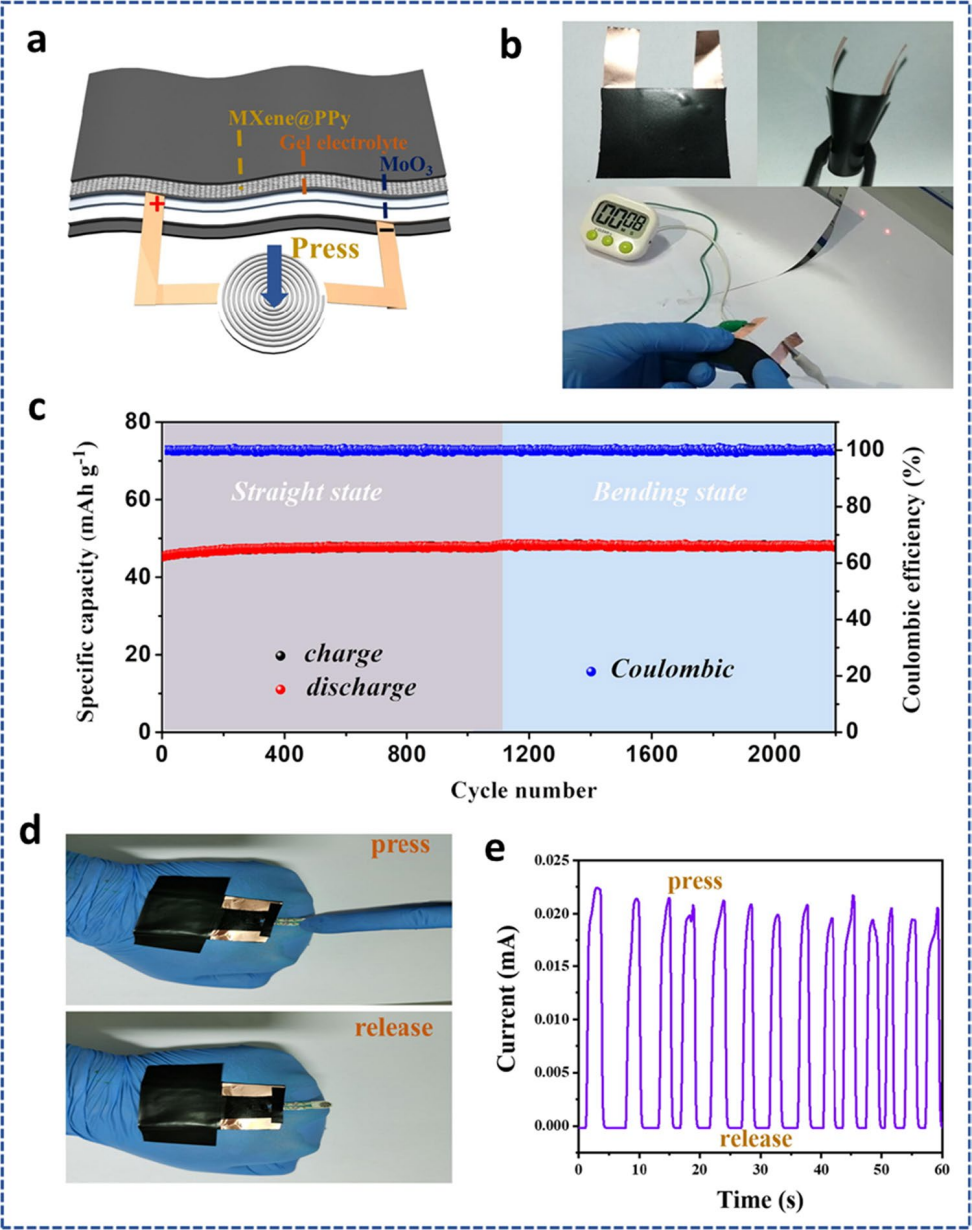
**a** Fabrication of the flexible RAB coupled with a pressure sensor. **b** Photographs of the power supply of flexible RAB under various bending conditions. **c** Cycle performances of flexible RAB in straight and bending states. **d** Pictures showing the real application of a pressure sensor integrated with the flexible RAB. **e** Sensing curves during the different pressures from the finger. Adapted with permission [192], Copyright (2022) by the authors. Published by Elsevier. This article is an open-access article distributed under the terms and conditions of the Creative Commons Attribution (CC BY) license

MXenes have adjustable features with their surface terminations, holding promise for a variety of applications including flexible batteries due to the great building blocks for the production of flexible films. For potassium-ion batteries (PIBs) and sodium-ion batteries (SIBs), hard carbon (HC) is a favorable anode material. However, the volume change caused by the insertion or extraction of K^+^ or Na^+^ limits the life cycle, particularly for PIBs because K^+^ has a large ionic size. Additionally, the coating process used to create traditional anodes cannot meet the demands of flexible devices. Sun et al*.* [191] demonstrated that 2D Ti_3_C_2_T_*x*_ carbide flakes can serve as versatile conductive binders for application as flexible HC electrodes. Hydrophilic and conductive 2D MXene nanosheets form 3D MXene-bonded networks of HC electrodes, which effectively stabilizes the electrode architecture and allows for the expansion of the volume of HC during the charge/discharge mechanism. This resulted in an increased electrode capacity and superb cycle performance as anodes for both PIBs and SIBs. The electrode film of the MXene-bonded HC exhibited an enhanced rate capability while gaining from the conductive 3D network, demonstrating that MXene is a very promising multi-facet binder for the next-generation flexible rechargeable batteries. Polypyrrole (PPy) has been identified as a good organic electrode for the application of rechargeable aqueous batteries (RABs); however, its rapid capacity deterioration after repeated cycling continues to limit its practical implementation. A multilayer composite of MXene-stabilized PPy (MXene@PPy) was purposefully designed using a simple dip-coating method [192]. Electrochemical measurements in conjunction with in situ Raman analysis displayed that the MXene coating effectively prevented the structural deterioration and irreversible redox mechanism of PPy during the electrochemical analysis. The synthesized MXene@PPy composite when used as a flexible RAB device (Fig. 23a, b), exhibited enhanced rate performance, long-term cycle stability, and 80.3% of the initial capacity retained over 2500 cycles. It also revealed high coulombic efficiency of 100% and excellent charge-transfer capability as shown in Fig. 23c–e. The enhanced results obtained from the flexible RAB can be attributed to the electron-rich and hole-rich regions created by the PPy and the MXene which facilitated the redox reaction of the synthesized composite.

**Fig. 24:**
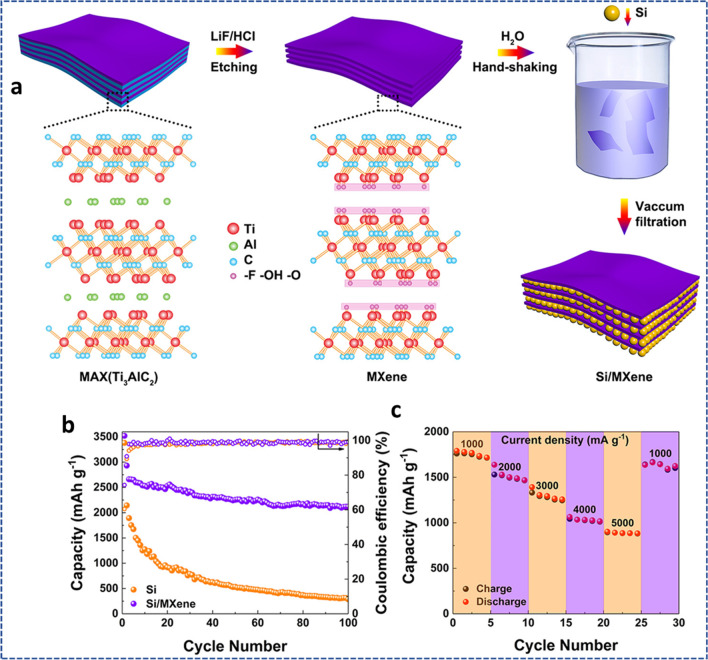
**a** Synthesis diagram for the Si/MXene composite paper. **b** Cycling stability of Si/MXene and pure Si anodes. **c** Rate ability of Si/MXene anodes at different current densities. Adapted with permission [194], Copyright (2019) American Chemical Society

Although 2D MXenes are promising materials for energy storage, their electrochemical performance is constrained by the restacking and aggregation of the 2D nanosheets. A 3D MXene foam with a designed porous structure was established using a straightforward sulfur-template technique to solve this issue and fully utilize the capabilities of MXene nanosheets [193]. This foam was flexible, freestanding, and highly conductive and was used as an electrode in flexible lithium-ion batteries. The MXene foam's 3D porous structure provided a significant amount of active sites to increase the capacity for lithium storage. Additionally, its foam shape made it easier for the electrolyte to infiltrate for quick Li^+^ transfer which contributed to the improved electrochemical performance. Due to the large theoretical capacity, low operating potential, and abundant supply, silicon has emerged as the anode choice that is most highly desired for lithium-ion batteries (LIBs). Its practical applicability is hampered by weak conductivity and significant volume growth. Flexible, freestanding, and binderless Si/MXene composite materials were created by vacuum filtration (Fig. 24a) and used as anodes for LIBs [194]. This novel structure exhibited excellent electrochemical performance with a high capacity of 2118 mAh/g at 200 mA/g after 100 cycles as shown in Fig. 24b,c. It also accommodated large volume expansion, higher conductivity of composites, and prevented restacking of MXene sheets.

## Summary and perspective

In recent years, stacked ternary metal nitrides, carbides, or carbonitrides have been selectively etched and exfoliated to yield more than 20 distinct 2D MXenes. MXenes easily produce a wide range of composites with other materials thanks to their great flexibility, layered structures, and 2D morphology, which provides an efficient way to tailor the chemistries and performances of MXenes for different applications. Adding CNTs and metal oxides as fillers to MXenes, for example, can improve their overall electrochemical performance by increasing their overall specific capacity and ability to conduct electrons and transport ions in the electrolyte.

This review discusses recent developments in the synthesis, characteristics, and applications of 2D MXenes and MXene-based composites. The most common synthesis techniques such as the top-down and bottom-up approaches for MXene have been discussed. Also, physical and chemical methods for synthesizing MXene nanocomposite materials have been carefully presented. In addition, the various properties (electronic, mechanical, and electrochemical) and how they can be modified for enhanced storage abilities have been highlighted. In particular, attention is paid to applications in electrochemical energy storage, such as supercapacitors, batteries, and their flexible components. MXenes and related composites are well suited for use in EES because of their exceptional characteristics, distinct morphologies, and layered structures. The research on MXenes is still in its early stages compared to that on graphene and other carbonaceous electrode materials. Future research on MXenes will face both opportunities and difficulties.

Considering the synthesis of MXene, while many MXenes, including Hf_2_C, Sc_2_C, W_2_C, and others, are forecast to be able to exist in a stable state, their precursors for synthesis have not yet been created. To increase the number of MXenes, it will be important to create new MAX phases or different layered nitride and carbide precursors. It would be ideal if novel techniques for producing high-quality MXenes with huge lateral dimensions, fewer flaws, and regulated surface terminations could be developed. The most difficult task for researchers is obtaining uniform terminations using just one kind of functional group. In the end, CVD or physical processes must be used to make pure MXenes with no surface terminations.

Most of the known characteristics in the area of property innovation are predicted by theoretical and computational calculations and are awaiting experimental confirmation. Additionally, there are no reports on the experimental measurements of the MXene band structures, which are crucial to comprehending the fundamental characteristics of MXenes. Understanding the structures and characteristics of intercalated MXenes with different ions/molecules is also essential. Although MXenes have demonstrated great performance in a variety of applications, numerous underlying physical principles still require extensive exploration, and the following points should be considered for the further development of MXene and MXene-based materials.

i. A thorough understanding of the electrochemical characteristics of diverse MXene and MXene-based composites is required for building novel, highly effective storage devices, which may also lead to new directions in the conversion and storage of sustainable energy.
ii. Future MXene research should combine computational and experimental studies. There are hundreds of possible MXene members when varied elemental compositions and surface terminations are taken into account. Scientists and researchers might be directed to synthesize and study the most interesting compounds using the previous computational predictions of the characteristics and potential applications of MXenes. This will considerably reduce the cost of the study. Machine learning has recently demonstrated its ability to correctly forecast the band gaps of various MXenes. In the near future, it is fair to anticipate that machine learning will play a considerable role in forecasting the characteristics of MXenes.
iii. The effectiveness of the electrochemical energy storage reaction is significantly influenced by the choice of structure. To research the electrochemical energy storage mechanism and further enhancements in performance, it is crucial to create synthesis processes to regulate the MXene’s surface and comprehend the structure–property relationship. Moreover, to enjoy the superior electronic and structural features of MXenes, researchers should focus on the construction of multifunctional MXene-based devices like MXene/polymer flexible devices and MXene-incorporated heterostructure-based smart devices.
iv. Moreover, from the point of view of precise fabrication and characterization of MXene-based materials, it is important to use in situ approaches (in situ SEM/TEM), and spectroscopic (Raman/Infrared) techniques can reveal the morphology and intermediate bonding as well as double-layer structure to the makeup of the efficient electrode materials. Notably, using these methodologies and doing analyses based on them can help fill in some significant knowledge gaps in this field. Moreover, to completely comprehend the development of the structure and composition of MXene electrodes, modern characterization approaches, synchrotron radiation systems, and solid-state nuclear magnetic techniques may also be helpful. This will help screen several MXene and MAX phases, comprehend the charge storage process, and examine the crucial connections between structure, property, and performance.

## Data Availability

As this is a review article, the datasets taken from the literature with proper copyrights are available and can be collected from the corresponding authors upon reasonable request.
